# Introducing the fusion innovation test as a new paradigm for studying realworld creative problem solving

**DOI:** 10.1038/s41598-025-28134-y

**Published:** 2025-12-23

**Authors:** Chien-Te Wu, Felix Benjamin Kern, Yu-Shiang Su, Miyoko Street, Zenas C. Chao

**Affiliations:** 1 International Research Center for Neurointelligence (WPI-IRCN), UTIAS, The University of Tokyo, Tokyo, Japan; 2 Department of Occupational Therapy, College of Public Health and Health Professions, University of Florida, Gainesville, FL USA; 3 Center for Cognitive Aging and Memory, McKnight Brain Institute, University of Florida, Gainesville, FL USA

**Keywords:** Creative problem-solving, Real-world creativity, Fusion innovation test, Alternative uses test, Kaufman domains of creativity scale, Creative achievement questionnaire, Human behaviour, Problem solving

## Abstract

Studying creative problem-solving requires tasks with real-world relevance and minimal domain-specific knowledge. The widely used alternative uses test (AUT) meets these criteria but primarily measures divergent thinking—generating many ideas without immediate judgment. However, creative problem-solving also requires convergent thinking to narrow down options and select the most effective solution. We introduce the fusion innovation test (FIT), a task that integrates both divergent and convergent thinking. In the FIT, participants combine two randomly assigned elements (e.g., objects or technologies) to achieve either a self-improvement goal or a sustainable development goal. Each FIT solution is evaluated based on the novelty and feasibility of combining the elements, and the effectiveness in achieving the intended goal. We collected behavioral data from 144 participants and used ratings from 122 human evaluators to train an automated scoring system based on a large language model. Participants also completed the AUT and two real-life creativity questionnaires. Results show that the FIT captures a unique aspect of creativity—goal-directed innovation—not assessed by the AUT. Moreover, FIT performance correlates with scientific creativity, whereas AUT performance aligns more with artistic creativity. These findings suggest that the FIT is an initially promising and complementary tool for studying real-world creative problem-solving.

**Supplementary Information:**

The online version contains supplementary material available at 10.1038/s41598-025-28134-y.

## Introduction

Creative problem-solving, the ability to provide novel and useful solutions to real-world problems, is an important part of human creativity^1,2^. Studying how we can improve our capacity of creative problem-solving has become an urgent need, especially considering the challenge by the advancement of artificial intelligence, e.g.^3–5^. To effectively assess creative problem-solving in a laboratory setting, a behavior paradigm should meet two key criteria. First, it should simulate real-world problem-solving by including a real-world goal. Second, to ensure broad applicability across diverse populations, it should require minimal domain-specific knowledge or skills. However, considering these criteria for a suitable behavior paradigm, options are limited.

From the perspective of simulating real-world problem-solving, many paradigms present tasks more like puzzle games that have less direct connection to everyday situations. Those include semantic puzzles such as the remote associates test (RAT)^6^, and anagrams^7^; association puzzles such as the Visual RAT^8^ and language-independent RAT^9^; numeric puzzles such as the hidden rule task^10^; drawing games such as the picture completion test^11–14^; spatial concept puzzles such as match problems^15^ and nine dot problems^16^.

On the other hand, paradigms that incorporate real-world goals^11–14,17–21^ often focus on specific knowledge or skills, making them more suitable for specialized populations. For example, some tasks require specialized engineering knowledge, such as engineer design problems^20,21^. Others emphasize design skills, like the product improvement test^13,14,19^ and the concept generation task for product designs^18^. Additionally, some tasks focus on drawing abilities, such as the book cover design task^17^.

Notably, the alternative uses test (AUT)^22^ and the realistic divergent thinking task^23–26^ stand out as appropriate paradigms that meet both criteria. The AUT challenges individuals to think of unusual uses for common objects, while the realistic divergent thinking task challenges individuals to come up with multiple ways to deal with a real-life situation. Both types of tasks frame their questions in real-life contexts and do not require extensive specialized knowledge or skills^27,28^. These tasks are typically used to study divergent thinking, the ability to generate many unique ideas or possibilities for a given quest^29^. Even though divergent thinking is a necessary component of creativity, creative problem-solving in everyday life often also requires us to narrow down options to pick the most effective solution with conditional limitations on available resources. This constraint-based option reduction involves convergent thinking, which is not typically emphasized in divergent thinking task. Therefore, there is a need for a new and complementary paradigm that also addresses the convergent thinking aspect of creativity.

In this study, we introduce the fusion innovation test (FIT) to assess both individuals’ divergent and convergent thinking when solving real-world problems using general imagination rather than domain-specific knowledge or skills. To ensure real-world relevance, the FIT requires participants to solve a real-world problem that is either related to a self-improvement goal (SIG), which addresses personal needs or well-being in daily life (e.g., reducing feelings of loneliness and isolation), or a Sustainable Development Goal (SDG), which relates to environmental or societal challenges (e.g., enhancing public awareness of land conservation). Furthermore, participants need to solve the problem with a constraint of combining two unrelated elements, which can be an object, a technology, a service, or a source of data. To ensure domain-generality, the problems and constraints are based on components typically encountered in everyday life. To enhance the applicability of the FIT, we provide the question sets in multiple languages (Japanese, English, and Chinese) and develop an automated evaluation method using a large language model, specifically GPT-4o. This GPT-based evaluation, similar to the one previously developed for AUT evaluation^30^, provides instant results without the need for human judges and does not require training data, making it highly convenient for users.

We collected behavioral data from 144 participants who engaged in the FIT, the AUT and two questionnaires: the Creative Achievement Questionnaire (CAQ)^31^ and the Kaufman Domains of Creativity Scale (K-DOCS)^32^. The two questionnaires were selected to measure individuals’ real-life creativity achievements and self-perceived creativity. The GPT ratings on FIT solutions were verified against ratings from 122 human raters, and later used to evaluate individual performances. We found that the GPT-rating method provides valid results, showing high correlations with human ratings. Furthermore, when correlated with the questionnaires, FIT performance indicates creativity in the science domain, whereas AUT performance indicates creativity in the art domain. Based on these results, we suggest that the FIT provides a suitable behavioral paradigm in the closed-ended problem-solving category, complementary to the AUT, to study human creativity.

## Methods

### Participants

We intended to conduct within-sample correlation analyses across various task performances, estimating that a minimum of 112 participants would be required to demonstrate a significant within-subject correlation. This estimate is based on the assumption of a minimal effect size |ρ|= 0.3, an type I error α of 0.05, and a power (1 − β) of 0.9, as calculated using G*power^33^. At the end, we collected 144 participants (101 males and 43 females, 22.8 ± 3.5 years old, mean ± std) participated the experiment. Additionally, 122 participants (82 males and 40 females, 23.1 ± 2.3 years old, mean ± std) served as human raters for the FIT evaluation. Both the experiment and the FIT evaluation were conducted on-site, under the guidance and supervision of experimenters, to ensure data quality. Participants were all native Japanese speakers recruited through a public website (https://www.jikken-baito.com). All participants signed informed consent before participation and were provided with monetary compensation after their participation. All study protocols were approved by the Research Ethics Committee at The University of Tokyo (No. 23-415) and all research was performed in accordance with relevant guidelines/regulations. Research involving human research participants was performed in accordance with the Declaration of Helsinki.

### Task materials

#### Fusion innovation test (FIT)

**Fig. 1:**
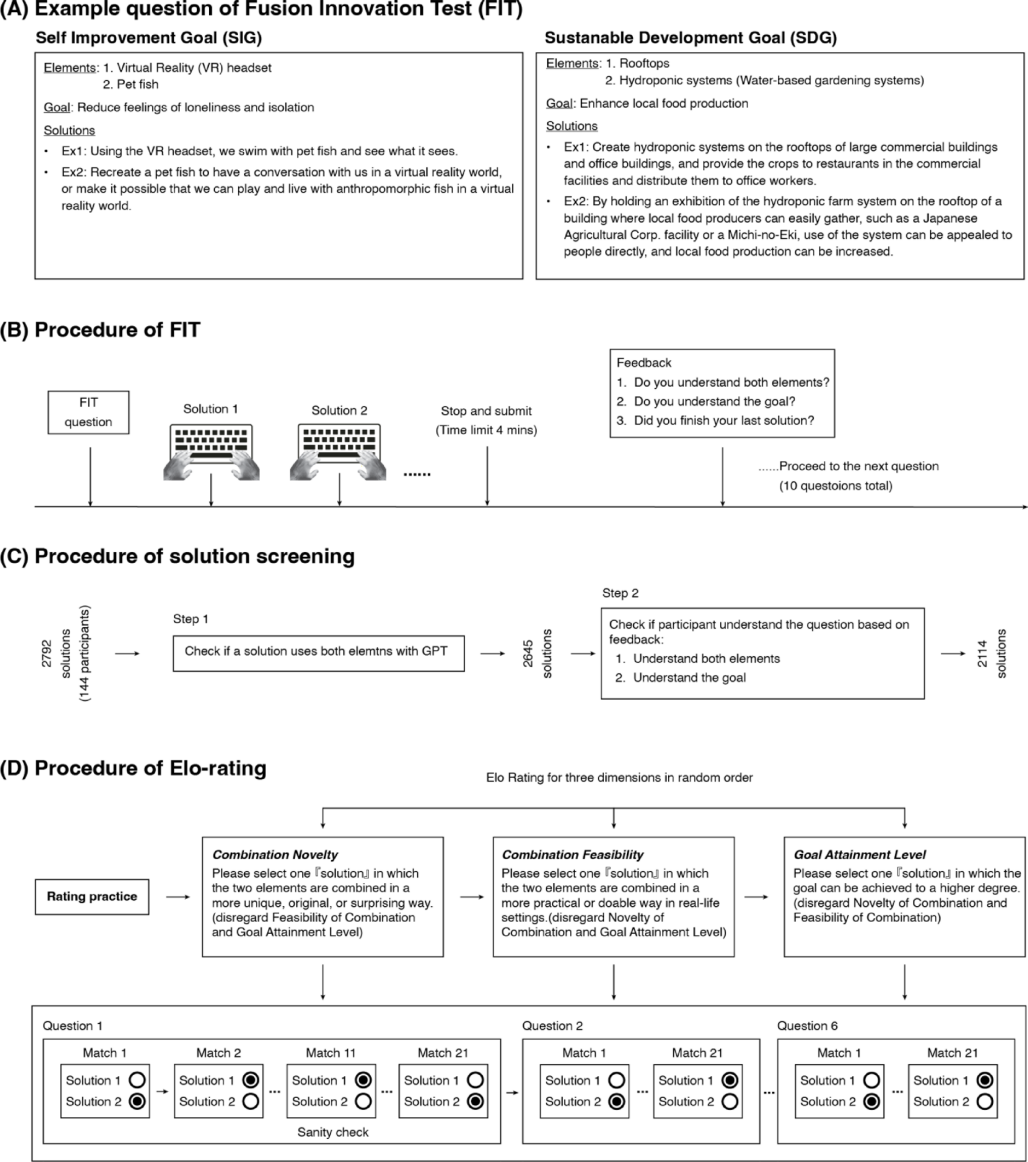
Materials, procedures, and evaluation of the Fusion Innovation Test (FIT). (**A**) Example questions and solutions for a Self Improvement Goal (SIG) and a Sustainable Development Goal (SDG). (**B**) For each question, participants were instructed to type as many creative solutions as possible within a time limit of 4 min. After submitting the answers, participants answered three feedback questions and then proceeded with the next question. (**C**) Screening for valid solutions with a 2-step process. Step-1: using GPT to ensure that both given elements were used in a solution. Step-2: using participant feedback to screen out solutions where participants denied comprehension of either elements or goal. (**D**) In Elo-rating, each rater performed evaluations on each dimension in random order, with each dimension containing 6 FIT questions and each question containing 21 matches. For each match, raters simply choose which solution has a higher score in the corresponding dimension. Furthermore, unbeknownst to raters, the 11th match was always the sanity check match which was used to control the quality of ratings.

Each FIT question is composed of two elements (e.g., virtual reality headset and pet fish) and one goal (e.g., to reduce feelings of loneliness and isolation), and participants are required to come up with creative solutions that integrate the two given elements to achieve the given goal. The FIT includes two types of goals: the Self Improvement Goal (SIG), and the Sustainable Development Goal (SDG) (Fig. 1A). The SIG focuses on improving personal needs or well-being in daily life, whereas the SDG focuses on addressing challenges faced by the environment or society. The current FIT includes 50 questions with 25 in the SIG category and 25 in the SDG category. The questions, including elements and goals, were first generated by GPT-4 and screened by experimenters (see details in Supplementary Methods). The English translations of the 50 questions are detailed in Table 1, while the original Japanese version can be found in Supplementary Table S1. We also provide a Chinese version in Supplementary Table S2. In our experiments, the 50 questions were divided into 5 sets with each set containing 5 SIG and 5 SDG questions (Table 1).

**Table 1:** English version of FIT.

FIT sets	Element 1	Element 2	Goal
Self improvement goal (SIG)			
1	Digital camera	Coloring book	Foster personal creativity and expression
1	Virtual reality (VR) headset	Pet fish	Reduce feelings of loneliness and isolation
1	Desk lamp	Water filter	Encourage regular hydration (water consumption)
1	Refrigerator	Allergy alert App	Assist in managing dietary restrictions
1	Padlock	Web browser	Protect personal digital privacy
2	Shopping cart	Barcode scanner	Simplify grocery shopping
2	Voice recorder	Language learning App	Enhance personal communication skills
2	Stress ball	Meditation App	Strengthen one’s capacity to endure and manage difficult times
2	YouTube	Puzzle	Assist in pursuing a new skill
2	Elevator	E-Library	Enable easier access to books or learning materials
3	Piggy bank	Spreadsheet software	Simplify personal budgeting and saving
3	Baby monitor	Parenting blog	Provide tools for effective parenting
3	Digital wallet	Review platform	Ensure a safe online shopping experience
3	Blender	Health and nutrition database	Strengthen immune system health
3	GPS device	Wardrobe closet	Improve your fashion choices and grooming routine
4	Webcam	Motion detector lights	Enhance home security
4	Hammock	Meditation App	Promote mental relaxation
4	Music streaming app (like Spotify)	Potted plant	Improve mental well-being
4	Filing cabinet	Smartphone reminder App	Improve personal space organization
4	Interactive quiz App	Microwave oven	Enhance learning opportunities outside of formal education
5	Vacuum cleaner	Calendar App	Make household tasks more efficient
5	Walking stick	Video calling device	Aid in the care of elderly family members
5	Smartphone	Medical journal	Enable access to quality healthcare information
5	Smartwatch	Pepper spray	Enhance personal safety
5	Public park bench	Social networking site	Encourage positive community engagement
Sustainable development goal (SDG)			
1	Electric cars	Travel review websites	Promote eco-friendly travel
1	Mobile alert App	Community radio station	Improve disaster preparedness
1	Parking lots	Ceramic filters	Create a rainwater collection system for effective water storage
1	Shipping containers	Green roof systems	Design affordable housing with minimal environmental impact
1	Cryptocurrencies	Job portals	Encourage equal pay for equal work
2	Animated series (animation production)	Weather station data	Foster education on climate change
2	Virtual reality (VR) conferences	University research labs	Promote collaborations to share sustainable technology
2	Coffee maker	Bamboo charcoal	Develop more effective ways to purify water for human consumption
2	Beeswax	Popsicle sticks	Propose innovative ideas for packaging that is eco-friendly
2	3D printers	Local artisanal crafts	To encourage eco-friendly product manufacturing in local community
3	Digital billboards	Drones	Enhance public awareness on land conservation
3	Virtual reality (VR) games	School curriculum	Encourage youth involvement in projects related to SDGs (Sustainable Development Goals)
3	Weather Apps	Doorbells with cameras	Design tools for early warning systems for natural disasters
3	Maglev train technology	Carpool App	Develop efficient public transportation systems
3	Algae	Underground metro systems	Design methods for carbon capture and storage
4	Wildlife documentary	Augmented Reality (AR) glass	Encourage biodiversity conservation
4	Library system	Refurbished laptops	Promote access to technology in impoverished communities
4	Exercise gym equipment	Solar panels	Design systems for clean energy generation
4	Vending machines	Recycling Apps	Design sustainable solutions for electronic waste
4	Fitness tracker	Local farmer’s market	promote health-related projects in impoverished communities
5	Rooftops	Hydroponic systems (Water-based gardening systems)	Enhance local food production
5	Air purifiers	Public buses	Enhance public health through clean air initiatives
5	International film festivals	Green technologies (environment-friendly)	Encourage international partnerships for SDGs (Sustainable Development Goals)
5	Bamboo fiber	QR code	Encourage sustainable fashion (Advocate for clothing that’s better for the environment)
5	E-readers	Solar-powered lanterns	Enhance digital literacy in impoverished communities

Set numbers refer to the question set used in the experiment.

#### Alternative uses test (AUT)

Each AUT question was composed of an everyday item (e.g., metal key), and participants were required to come up with creative alternative uses for the given item. Similar to the FIT, our version of the AUT contains a pool of 50 everyday items which were also divided into 5 sets of questions with each set containing 10 questions (Table S3).

#### Creative achievement questionnaire (CAQ)

The CAQ is a 96-item self-report instrument that evaluates a participant’s achievement in the following 10 creativity-related domains: visual arts, music, dance, creative writing, architectural design, humor, theater and film, culinary arts, inventions, and scientific inquiry^31^. The evaluation primarily relies on public recognition records of the participant’s creative works, such as earning awards in competitions or presenting works in public exhibitions, within the relevant domain. The CAQ scores can be viewed as an objective measure of creativity achievement in real life.

#### Kaufman domains of creativity scale (K-DOCS)

The K-DOCS is a 50-item self-report questionnaire, which uses a 5-point Likert scale to evaluate the subjective perception of self-creativity as compared to the general population in the following 5 domains: self/everyday, scholarly, performance, mechanics/scientific, artistic^32^. The K-DOCS scores can be viewed as a subjective measure of self-perceived creativity in real life.

### Task procedures

The experiment was implemented through a custom-made website on which each participant performed the tasks in the following order: a set of 10 FIT questions, K-DOCS, CAQ, and then a set of 10 AUT questions. The order of questions within each FIT and AUT set was randomized. Furthermore, the combination of FIT and AUT question sets were also pseudo-randomly assigned so that all possible combinations of a FIT set and an AUT set were equally distributed, without any question-set combination bias between FIT and AUT.

#### FIT procedure

For each FIT question, participants were required to generate as many creative solutions as possible within a 4-min time limit (Fig. 1B). The 4 min was decided based on internal testing where people can comfortably generate at least 2 solutions for FIT questions. They were instructed to type each solution in a comprehensive short paragraph in an input box. There were two input boxes shown on the screen to start with. When these two boxes were filled, a new input box would automatically appear for participants to type into. A timer was displayed to remind participants about the remaining time. When the remaining time was 30 s or less, the timer started to flash to remind participants that the question would terminate soon. Once the time limit was reached, all input boxes were locked from typing and participants had to submit the solutions. Following each trial, participants answered three feedback questions: ‘(1) Do you understand both elements?’, ‘(2) Do you understand the goal?’, and ‘(3) Did you complete the final solution when the time was over?’. The feedback from the 3 questions was used to filter out invalid solutions from analysis. After answering feedback questions, they clicked a button to proceed to the next FIT question.

#### AUT procedure

For each AUT question, participants were required to generate as many creative alternative uses as possible for the given item within a 1.5-min time limit. The 1.5 min was decided based on internal testing where people can comfortably generate at least 2 solutions for AUT questions. They were instructed to type each alternative use in a comprehensive short sentence in an input box. There were three input boxes shown on the screen to start with. When these three boxes were used, a new input box would automatically appear for participants to type into. A timer was displayed to remind participants about the remaining time. When the remaining time was 30 s or less, the timer started to flash to remind participants that the trial would terminate soon. Once the time limit was reached, all input boxes were locked from typing and participants had to submit the solutions and proceed to the next AUT question.

#### CAQ and K-DOCS procedures

For the CAQ, the 10 subscales were presented in a fixed order with each subscale containing 8 items. Items that are a description (e.g., “I have played with a recognized orchestra or band”) were presented with a yes-or-not checkbox mechanism, while for items asking about the frequency (e.g., “My work has been critiqued in national publications.”), participants were asked to write the number of times this sentence applies. For the K-DOCS, the 50 items were presented in a randomized order. For each item (e.g., “Writing a poem”), participants needed to rate themselves on a Likert scale from 1 to 5 (1 = Much less creative, 2 = less creative, 3 = neither more nor less creative, 4 = more creative, 5 = much more creative).

### Task evaluations

#### FIT evaluation

The FIT was evaluated in 4 dimensions. For each FIT question, we measured the *Fluency*, which is the total number of proposed solutions. Furthermore, each solution was evaluated across three additional dimensions: *Combination Novelty*, *Combination Feasibility*, and *Goal Attainment Level*. The first two dimensions focus exclusively on how the two question elements are utilized, while the last dimension solely evaluates whether the goal is satisfactorily achieved. Specifically, Combination Novelty of a solution assesses how unique or surprising it is for the two elements to be combined or integrated as described, Combination Feasibility of a solution assesses how practical or doable in real-life it would be to combine or integrate the two elements as described, and Goal Attainment Level assesses the degree to which a proposed solution achieves the given goal.

Our objective was to develop an automated FIT evaluation across these three dimensions using a large language model, specifically GPT-4o, similar to our GPT-based AUT evaluation^30^. This method offers instant results without the need for multiple human judges. Additionally, our approach does not require training data, making it convenient for potential users of the FIT. In order to verify the prompt designs for GPT, we compared them to the ground truth, which we acquired from 122 human raters using an Elo-rating approach (see below).

To facilitate effective Elo-rating and GPT evaluation, we first screened out invalid solutions with a 2-step procedure (Fig. 1C). In Step 1, we checked whether a solution used both elements using the following GPT prompt: ”*In the scenario "{Solution}", the two elements "{Element_1}" and "{Element_2}" both play a role: Yes/No*”. We conducted this check for each solution three times, and only solutions receiving three “yes” votes were advanced to the next step. In Step 2, solutions that passed the Step 1 check (2645 out of 2792 solutions from 144 participants) underwent further screening. Any solution where either the elements or the goal were unclear to a participant (based on the after-question feedback) was eliminated. Subsequently, 2114 valid solutions remained.

#### Elo-rating for FIT solutions

To establish ground-truth ratings for all valid solutions from human raters, we used an Elo-rating approach^34^, which has been implemented for the AUT^30^. Specifically, raters evaluated the Combination Novelty, Combination Feasibility and Goal Attainment Level for solutions in a pairwise match context. In each match, they performed a two-alternative forced choice and judged which of the two solutions for the corresponding FIT question is better in one of the three dimensions (e.g., higher Combination Novelty).

Each rater performed evaluation on all three dimensions in random order, with each dimension containing 6 FIT questions (Fig. 1D, top row). The raters underwent 2 practice matches for each dimension before they started. For each FIT question, they evaluated 21 pairs of solutions for the given question, with the 11th pair being a sanity-check pair (unbeknownst to the raters) (Fig. 1D, bottom row). The selection of the solution pair for a match was pseudo-randomly assigned with higher priority to select two solutions with closer ratings in the given dimension. The sanity check pair contained solutions designed to have obvious win-loss judgment. For example, for the FIT question with “digital camera” and “coloring book” as the two elements and “foster personal creativity and expression” as the goal, the sanity check pair for Combination Novelty was to compare “Develop a digital camera that converts photos into coloring book templates, allowing users to print and color their own photographs, blending photography with traditional art.” and “Take photos with a digital camera and use them as references for illustrations to be manually traced into a coloring book.” In this case, the former clearly shows a more novel way to combine the two given elements. The sanity check pairs were generated using GPT with structured prompts (for the detailed prompts please see Supplementary Methods) and were internally checked and consensually agreed-upon by three authors. The sanity check pair was inserted as a quality control to assure that participants were not simply randomly picking winners. Furthermore, motivational text messages (e.g., ‘you are doing a good job, keep going!’, or ‘Try to read it carefully, if you have any questions, please ask the investigator’) would popout following each sanity check question to help maintain high levels of engagement of participants. Any results from a set that did not pass the sanity check were not included for updating the ratings. On average, each solution was compared 12.64 ± 5 times (mean ± std, 6–47 times) for Combination Novelty, 11.84 ± 4.64 times (mean ± std, 5–41 times) for Combination Feasibility, and 13.28 ± 6.31 times (mean ± std, 6–55 times) for Goal Attainment Level. The histograms of match counts for all solutions within each dimension are summarized in Supplementary Fig. S1.

Following the procedures, the solution ratings were determined based on their match results with other solutions. Specifically, the Elo-rating scores for any solution in any dimension were determined in two steps^34^. In the first step, for a pair of solutions A and B from a FIT question, we calculated the expected odds of winning, which are denoted as ${E}_{A}$ and ${E}_{B}$ respectively, with the following equations:

1 $${E}_{A}=\frac{1}{1+{10}^{\left({R}_{B} -{R}_{A} \right)/400}}$$

2 $${E}_{B}=\frac{1}{1+{10}^{\left({R}_{A} -{R}_{B} \right)/400}}$$

Here, R_A_ and R_B_ denote the initial ranking scores of A and B, respectively, before entering the match. In the second step, based on *E*_*A*_ and *E*_*B*_ and the match result, we can then calculate the new ranking scores ${{R}^{\prime}}_{A}$ and ${{R}^{\prime}}_{B}$ as follows:

3 $${{R}^{\prime}}_{A}={R}_{A} + K ({S}_{A} - {E}_{A})$$

4 $${{R}^{\prime}}_{B}={R}_{B} + K ({S}_{B} - {E}_{B})$$

Here, ${S}_{A}$ and ${S}_{B}$ denote the match outcomes with 1 referring to a win and 0 referring to a loss. $K$ denotes the K-factor, a score adjusting constant, and is set to be 16 in the current rating system as in our previous study^30^. The initial scores for all solutions were typically set to be 1600. By running the algorithm iteratively through all match results, we obtained corresponding Elo-ratings for all solutions in Combination Novelty, Combination Feasibility and Goal Attainment Level for each FIT question.

There are two primary characteristics of the Elo-rating algorithm. First and most intuitively, a solution with more wins will have a higher score. Second, winning or losing against higher-ranking solutions yields a greater rank reward or deduction, respectively, and vice versa for against lower-ranking solutions. The ratings of all solutions were updated every 30 min, and the updated ratings were used to assign the matches for the next round for a human evaluator.

#### GPT-rating for FIT solutions

We developed an API-based pipeline that systematically fed prompts and data to GPT-4o for rating the performance of FIT solutions. The prompts were designed with 5 key principles suggested and verified in^30^: (1) Ratings were implemented independently for only one dimension, without considering the other two. (2) Solutions from the same question were rated in batch mode, with 20 solutions per batch. (3) The rating scale ranged from 1 to 100, with 1 as the lowest score and 100 as the highest. (4) We asked GPT-4o to provide elaborations on the solutions and rating rationales before providing the actual ratings (“explain first, rate later” design). (5) All solutions were randomly shuffled before being input into GPT-4o.

For each solution, we implemented the rating procedures three times for each dimension and extracted the average score. Table 2 lists all the prompts, which contain 4 building blocks including Introduction (describing the FIT), Dimension (defining the evaluation scope for the corresponding dimension), FIT question, and Solutions. In implementation, prompts were fed into GPT-4o in English. To provide a more comprehensive picture about the effectiveness of the GPT-rating approach in different language contexts, we specifically compare GPT performance between the following two scenarios: rating with FIT questions and solutions in participants’ native language (Japanese) versus the original language of GPT (English). To achieve this, we used GPT-4o to do a batch process that translates all Japanese solutions into English (see details in Supplementary Methods).

**Table 2:** GPT prompts for solution evaluation of FIT.

Building block		Prompt
Introduction		We aim to evaluate the creativity of solutions in the Fusion Innovation Test (FIT) based on specific criteria. In each question of FIT, people will be presented with 2 elements and 1 goal, and they are required to come up with creative solutions that combine the 2 provided elements to achieve the given goal. In a solution, the 2 given elements must be used, but they were allowed to add additional elements into their solutions whenever needed
Dimensions	Combination novelty	Please rate these solutions, given below, in terms of the Novelty of the combination of 2 elements in solutions, which is defined as follows: Rate the novelty of the combination of 2 elements in a solution on a scale of 1–100, with 1 being not novel at all and 100 being extremely novel. Consider how unique, original, or surprising the two given elements are combined in a solution, while disregarding the feasibility of the combination of 2 elements and goal attainment level in that solution. Make sure to give a rating to every solution, disregarding the quality of the sentence Proceed as follows in your evaluation Write 3 (three) lines for each solution in the list below. On the first line, write the solution number and briefly describe the solution in your own words. On the second line, consider other novel ways to combine the two elements, including those listed, and compare the solution to these in terms of its Novelty of the combination of 2 elements. Finally, on the third line, provide your numeric rating as a json object of the form {“novelty”:x}. Evaluate each solution in the order provided, leaving one empty line between evaluations. Do evaluate each of the 20 solutions below individually and make sure to give a rating to every solution, even if there are repetitions
	Combination feasibility	Please rate these solutions, given below, in terms of the Feasibility of the combination of 2 elements in solutions, which is defined as follows: Rate the feasibility of the combination of 2 elements in a solution on a scale of 1–100, with 1 being not feasible at all and 100 being extremely feasible. Consider how practical or doable in real-life the way two elements are combined in the solution, while disregarding the novelty of the combination of 2 elements and goal attainment level in the solution. Make sure to give a rating to every solution, disregarding the quality of the sentence Proceed as follows in your evaluation Write 3 (three) lines for each solution in the list below. On the first line, write the solution number and briefly describe the solution in your own words. On the second line, consider other feasible ways to combine the two elements, including those listed, and compare the solution to these in terms of its Feasibility of the combination of 2 elements. Finally, on the third line, provide your numeric rating as a json object of the form {“feasibility”:x}. Evaluate each solution in the order provided, leaving one empty line between evaluations. Do evaluate each of the 20 solutions below individually and make sure to give a rating to every solution, even if there are repetitions
	Goal attainment level	Please rate these solutions, given below, in terms of their Goal Attainment Level, which is defined as follows: Rate the goal attainment level of the solution on a scale of 1 to 100, with 1 being not achieving the goal at all and 100 being achieving the goal with an extremely high level. Consider to what level the solution achieves the given goal, while disregarding the novelty and feasibility of the combination of 2 elements in the solution. Make sure to give a rating to every solution, disregarding the quality of the sentence Proceed as follows in your evaluation Write 3 (three) lines for each solution in the list below. On the first line, write the solution number and briefly describe the solution in your own words. On the second line, consider other solutions with a high level of goal attainment, including those listed, and compare the solution to these in terms of its Goal Attainment Level. Finally, on the third line, provide your numeric rating as a json object of the form {“goal attainment level”:x}. Evaluate each solution in the order provided, leaving one empty line between evaluations. Do evaluate each of the 20 solutions below individually and make sure to give a rating to every solution, even if there are repetitions
FIT question		FIT question Elements (1) デジタルカメラ (2) 塗り絵本 Goal 個人の創造性と表現を培う
Solutions		(1) 塗り絵本で塗った絵をデジタルカメラで取り込み、さまざまな加工ができるアプリを介して絵を色々な表現で加工できるようにする。 (2) デジタルカメラで撮ったものをAI技術で線画にし、塗り絵本としてタブレット等で用いれるようにする。 (3) VR、AR技術を用いて塗り絵本の世界に没入できるようにし、デジタルカメラでのさまざまな角度での撮影ができ、追加工するようにする。 (4)…..[continuously list until 20 solutions]

#### AUT evaluation

Before evaluation, we first screened out invalid solutions by filtering out any solution that was identified as an incomplete sentence using GPT-4o with following steps: All AUT answers were screened for its completeness with the following steps. Step 1: an AUT answer was identified as a complete answer if the answer ended with a “$\circ$” symbol (the period symbol in Japanese texts). Step 2: an AUT answer consisting of just a repetition of the question item per se was identified as incomplete (e.g., question: umbrella, answer: umbrella). Step 3: the remaining answers (n = 2718) were queried through GPT-4 (model: gpt-4-1106-preview) with the prompt “*The string “{Answer}” is a participant’s answer to the question of alternative uses of the item “{Question}”. Input was cut off after a time limit. Does the answer, which may be extremely brief, appear to have been cut off? Yes/No*”. If the response started with “No”, the answer was identified as complete.

All valid answers of AUT were then evaluated in 3 dimensions. For each AUT question, we measured the *Fluency* (the total number of proposed solutions). Furthermore, each solution was evaluated across two dimensions: the *Novelty* and *Feasibility* of completed ideas by a GPT-based approach^30^. Specifically, Novelty assesses how unique, original, or surprising an alternative is, while disregarding its feasibility. Feasibility assesses how practical or doable an alternative use is in real-life settings, while disregarding its novelty. Details on the prompt design can be found in^30^.

#### CAQ and K-DOCS evaluations

The CAQ and K-DOCS scores were both summarized into the science and art domain scores for further analyses. For the CAQ, following the suggestion based on a factor analysis from the developer^31^, we aggregated the scores from the 10 subscales into two domain measures with CAQ-Science to include subscales of culinary arts, inventions, and scientific inquiry and CAQ-Art to include subscales of visual arts, music, dance, creative writing, humor, and theater and film. The subscale of architectural design was removed from analysis due to the low factor loading and frequent low scores or zero scores from a younger population^31^. For the K-DOCS, based on a factor analysis showing a two-factor loading characteristic from the developer^35^, we aggregated the scores to form two domain measures with K-DOCS-Science to include the subscales of self/everyday, scholarly, and mechanics/scientific, and K-DOCS-Art to include subscales of performance and artistic.

### Data analysis

#### Verifying GPT-rating performance

To verify the GPT-rating performance, we first calculated the weighted rank correlation^36^ between the GPT ratings and Elo ratings across all valid solutions for each FIT question, with the match counts of the Elo-ratings as the corresponding weights. This approach helps reduce the potential unequal reliability due to different match numbers in Elo ratings between solutions. The GPT-rating performance was then determined by the average correlation coefficient *r* across all 50 questions. The violine plots were implemented by the MATLAB code ‘violineplot.m’ provided by Bastian Bechtold (https://github.com/bastibe/Violinplot-Matlab). The computation of Cohen’s d was implemented by the MATLAB code ‘computeCohen_d.m’ provided by Ruggero G. Bettinardi (https://github.com/Abolfazl-Alipour/BottomUP-TopDown-InfoFlowInNeuroDegeneration). The Bayes Factor (BF_10_) for the comparisons between GPT-rating performances was calculated using a MATLAB-based toolbox^37^.

#### Characteristics of GPT scores of FIT evaluation dimensions

To delineate the characteristics of the GPT scores of all 3 evaluation dimensions in FIT, we first plot the histogram of each dimension and calculate the corresponding skewness (skewness.m in MATLAB). We then further quantify the relationship between the 3 dimensions with Spearman’s ranked correlation.

#### Quantifying individual performance

For the FIT, individual performance was evaluated by the average scores across questions in Fluency, Combination Novelty, Combination Feasibility, and Goal Attainment Level, resulting in 4 scalar values. Specifically, performances in Combination Novelty, Combination Feasibility and Goal Attainment Level were measured by the average across questions of the highest scores across all solutions within each question in the corresponding dimension. For the AUT, individual performance was evaluated by the average scores across questions in Fluency, Novelty, and Feasibility, resulting in 3 scalar values. Specifically, performances in Novelty and Feasibility were measured by the average across questions of the highest scores across all solutions within each question in the corresponding dimension. The rationale of using the highest score within a question was that we were interested in the “best” performance a participant can achieve within the time limit given the task instruction where they were asked to generate as many creative solutions/alternative uses as possible. Such scenario mimics the real-world situation where we want to identify the “best” idea during a brainstorming process. Note that for both FIT and AUT, all scores except Fluency were extracted using the GPT-rating approach.

#### FIT vs. AUT, CAQ and K-DOCS

To examine the key features of the measurement dimensions in the FIT, we performed Spearman’s ranked correlation analyses on individual performances between the four dimensions in the FIT and between the four dimensions in the FIT and the three dimensions in the AUT. Furthermore, to evaluate how well FIT performances inform about individual real-life creativity, as compared to the AUT, we performed Spearman’s ranked correlation analyses between the performances in the FIT and the AUT and the scores of the CAQ and the K-DOCS.

## Results

### GPT-rating on FIT solutions

**Fig. 2:**
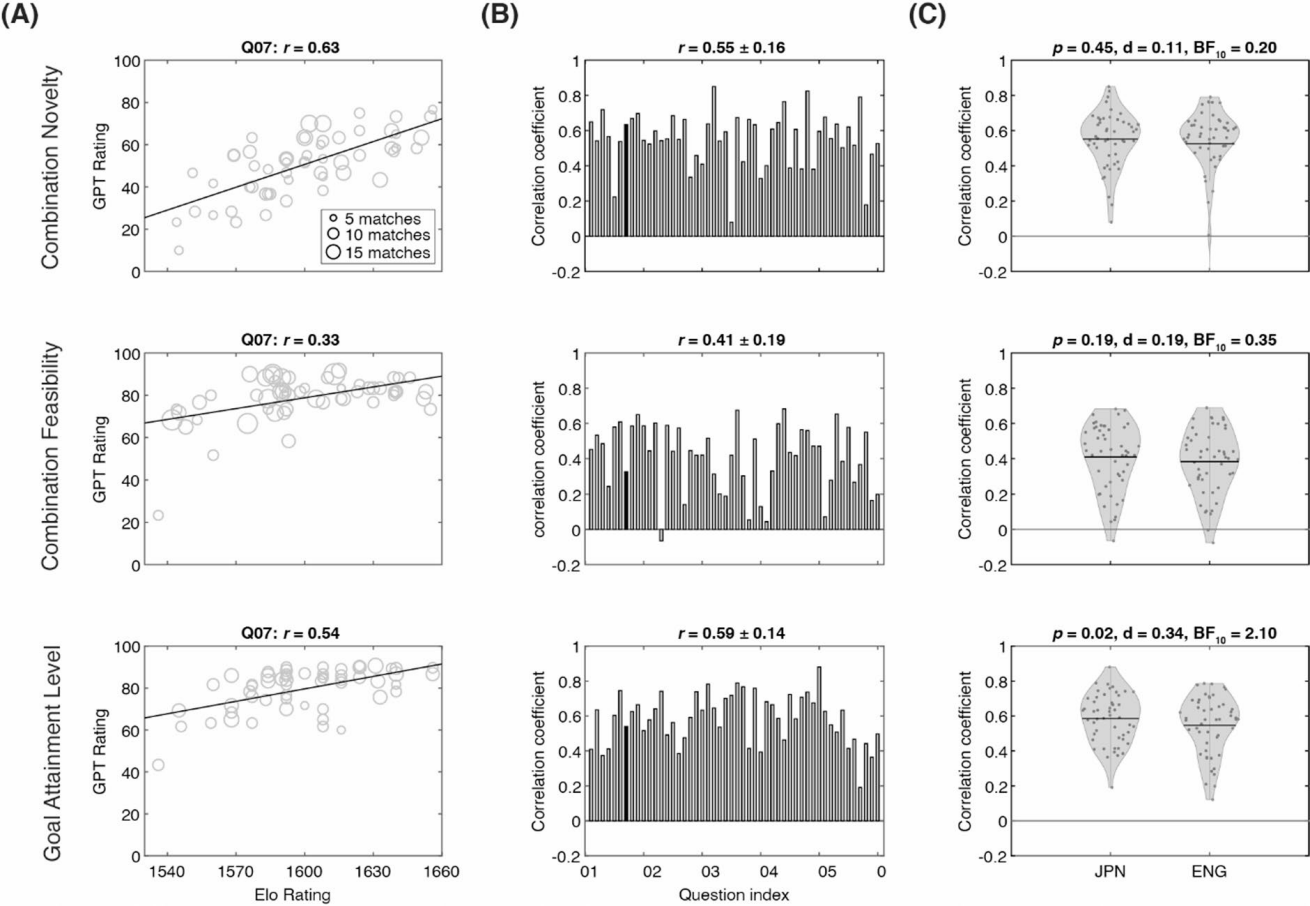
Summary of GPT-rating performances on FIT solutions. (**A**) The scatter plot of the scores from GPT-rating (y-axis) against Elo-rating (x-axis) for Question No. 7 for Combination Novelty (top), Combination Feasibility (middle), and Goal Attainment Level (bottom). The sizes of circles represent the corresponding match counts in Elo-rating. Linear regression lines are shown for visualization purposes. The corresponding weighted rank correlation coefficient *r* is shown. (**B**) The bar plots show *r* values across all 50 questions for Combination Novelty (top), Combination Feasibility (middle), and Goal Attainment Level (bottom), under the Japanese context. The corresponding mean and standard deviation of the 50 weighted correlation coefficients *r* are provided on the top of each bar graph. The black bars indicate the values for Question No. 7 as shown in panel A. (**C**) Violin plots of the distribution of 50 *r* values under the Japanese (JPN) and English (ENG) contexts for Combination Novelty (top), Combination Feasibility (middle), and Goal Attainment Level (bottom). The black solid lines in each plot represent the means. The results of statistical tests between two language contexts are provided on the top of each violin plot. The corresponding correlation coefficient *r* (mean ± standard deviation) is shown. Note. d = Cohen’s d; BF_10_ = Bayes Factor.

Figure 2A illustrates an example of the relationship between Elo-rating and GPT-rating for a FIT question (Question #7), where the GPT-rating were based on Japanese (not English translations). The corresponding weighted rank correlation coefficients were 0.63, 0.33, and 0.54 for Combination Novelty, Combination Feasibility, and Goal Attainment Level, respectively (Fig. 2A). The weighted rank correlation coefficients for all 50 questions are plotted in Fig. 2B, and the average correlation coefficients were 0.55 ± 0.16, 0.41 ± 0.19, and 0.59 ± 0.14 (mean ± standard deviation, n = 50 questions) for Combination Novelty, Combination Feasibility, and Goal Attainment Level, respectively. On the other hand, for GPT-rating based on English translations, the average correlation coefficients were 0.53 ± 0.27, 0.38 ± 0.18, and 0.55 ± 0.17 for Combination Novelty, Combination Feasibility, and Goal Attainment Level, respectively (Fig. 2C). There was no significant difference between GPT-rating based on Japanese and English translations for Combination Novelty (*p* = 0.45, Cohen’s d = 0.11, BF_10_ = 0.20) and Combination Feasibility (*p* = 0.19, Cohen’s d = 0.19, BF_10_ = 0.35). However, for Goal Attainment Level, GPT-rating based on Japanese offered significantly higher performance than the one based on English translations (*p* = 0.02, d = 0.34, BF_10_ = 2.10). This suggests that translating questions and answers into English does not improve GPT-rating performance. Therefore, we used Japanese for GPT ratings in subsequent analyses of the FIT. We selected this approach not only because of its slightly better overall performance but also due to its straightforward applicability, allowing the FIT to be provided and assessed in its original language without the need for translation.

### Three dimensions of the FIT

**Fig. 3:**
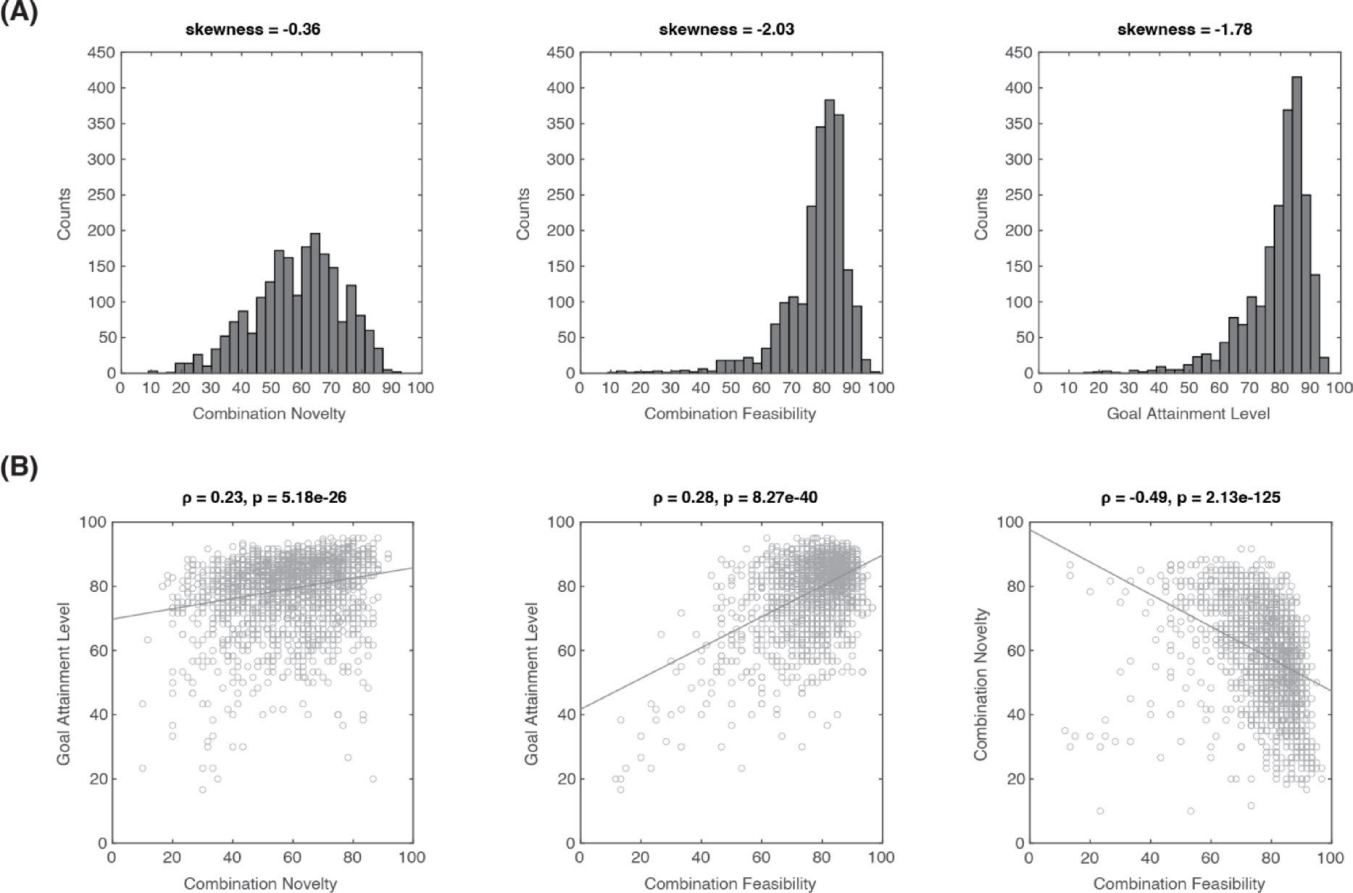
Distributions of each measurement dimension and correlations between measurement dimensions within the FIT. (**A**) The histograms of score distribution of Combination Novelty, Combination Feasibility, and Goal Attainment Level. The corresponding skewness values are provided at the top of each histogram. (**B**) Scatter plots of all solutions (2114 valid solutions from 144 participants) for Goal Attainment Level vs. Combination Novelty, Goal Attainment Level vs. Combination Feasibility, and Combination Novelty vs. Combination Feasibility. The corresponding Spearman’s ρ and p-values are provided at the top of each plot. The solid lines represent the linear regression lines (for visualization purpose only).

First, we plot the score distribution of all solutions for each evaluation dimension in FIT: Combination Novelty, Combination Feasibility, and Goal Attainment Level. As shown in Fig. 3A, the distribution of Combination Feasibility (skewness = − 2.03) and Goal Attainment Level (skewness = − 1.78) showed a left skewness while the distribution of Combination Novelty (skewness = − 0.36) is rather symmetric. This pattern aligns with the FIT’s design to focus on solving a specific goal, leading participants to prioritize feasible solutions over novelty. A similar phenomenon was observed in the AUT, where participants focused on “use” before “alternative” and prioritized feasibility over novelty (see Supplemental Fig. S2).

To further assess the relationships among the three dimensions in FIT, we analyzed the correlations between them across all 2114 solutions provided by the 144 participants (Fig. 3B). Our results indicate that the Goal Attainment Level positively correlated with Combination Novelty (Spearman’s ρ = 0.23, *p* = 5.18e−26) and Combination Feasibility (ρ = 0.28, *p* = 8.27e−40). This indicates that solutions with higher goal attainment are linked to more unique and practical combinations of elements. On the other hand, a negative correlation was found between Combination Novelty and Combination Feasibility (ρ = − 0.49, *p* = 2.13e−125). This is consistent with the notion that original solutions are often less practical.

### Individual performance in the FIT

**Fig. 4:**
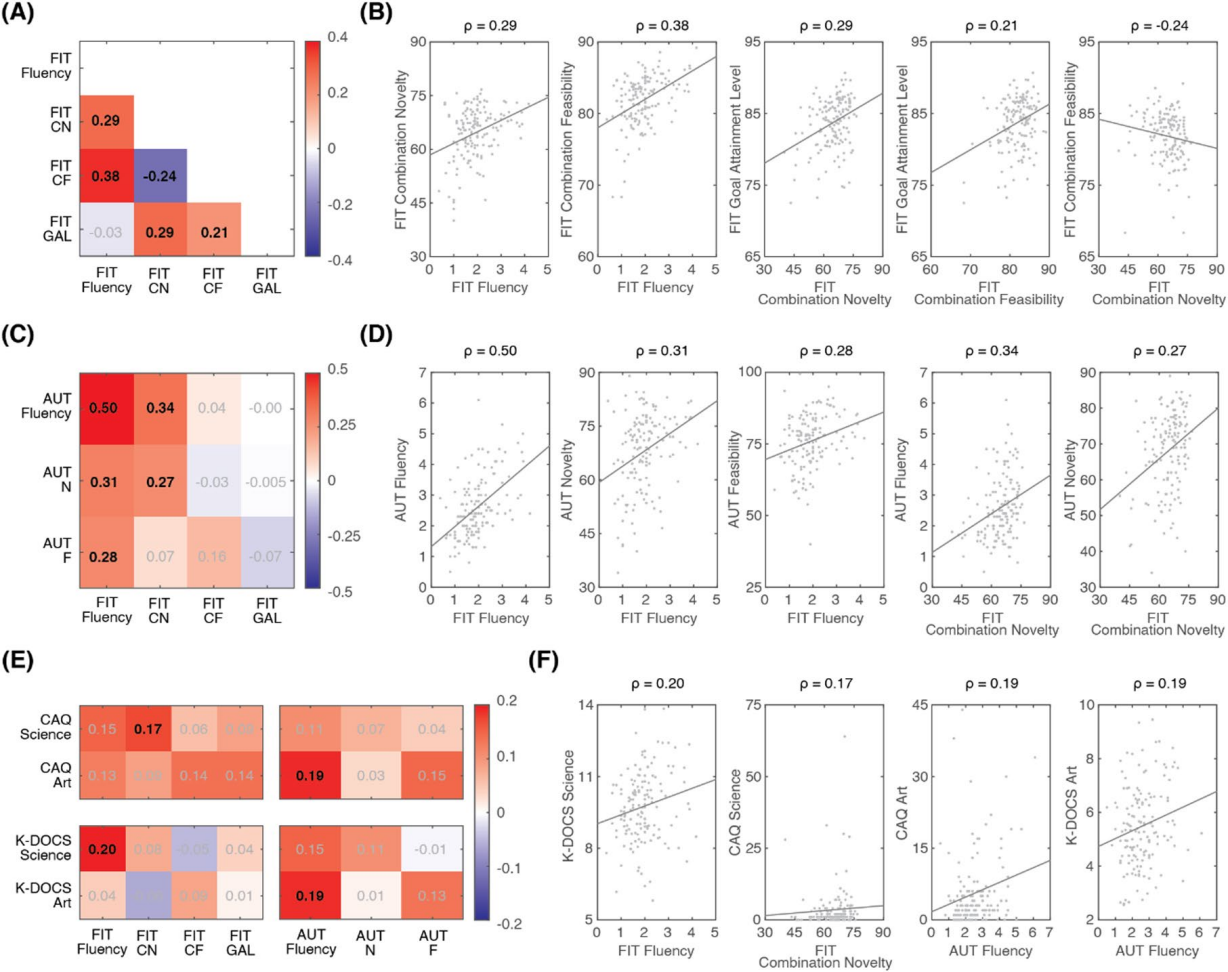
Correlations in individual performance across measurement dimensions of FIT, AUT, CAQ, and K-DOCS. (**A**) Correlation matrix between measurement dimensions within the FIT, and (**B**) the corresponding data distribution for significant correlations. (**C**) Correlation matrix between measurement dimensions of the FIT and the AUT, and (**D**) the corresponding data distribution for significant correlations. (**E**) Correlation matrix between measurement dimensions of FIT/AUT and CAQ/K-DOCS, and (**F**) the corresponding data distribution for significant correlations. Color indicates the strength of correlation as calculated by Spearman’s ρ, with red representing positive correlation and blue representing negative correlation. Significant correlations are highlighted in black and bold text. The solid lines in scatter plots represent the linear regression lines (for visualization purpose only). Note. For the FIT, CN = Combination Novelty, CF = Combination Feasibility, GAL = Goal Attainment Level. For the AUT, N = Novelty, F = Feasibility.

To evaluate individual performance, we assessed both the quality of the solutions (through the three FIT dimensions) and the quantity of solutions (Fluency). Figure 4A summarizes the correlations among the average values of these four measures across participants. Similar to the correlations across solutions, analyses across participants revealed that Goal Attainment Level was positively correlated with both Combination Novelty (Spearman’s ρ = 0.29, *p* = 0.00043) and Combination Feasibility (ρ = 0.21, *p* = 0.013), whereas a negative correlation was observed between Combination Novelty and Combination Feasibility (ρ = − 0.24, *p* = 0.0037). In addition, Fluency was positively correlated with both Combination Novelty (ρ = 0.29, *p* = 0.00046) and Combination Feasibility (ρ = 0.38, *p* = 2.62e−6), but not with Goal Attainment Level (ρ = 0.03, *p* = 0.71). This suggests that individuals capable of generating a greater number of solutions tend to demonstrate more creative ways of combining elements, consistent with findings related to divergent thinking^38^. The individual values for these significant correlations are further shown in Fig. 4B.

### Correlation between the FIT and the AUT

Next, we examined the correlation between individual performance in the FIT and performance in the AUT. The results are summarized in Fig. 4C (the individual values for significant correlations are further shown in Fig. 4D). The goal here was to determine whether the FIT and the AUT provide similar assessment of individual creativity, and to identify any unique contributions each test may offer.

First, Fluency in the FIT showed significant positive correlation with Fluency in the AUT (Spearman’s ρ = 0.50, *p* = 1.8e−10). Also, Combination Novelty in the FIT showed significant positive correlation with Novelty in the AUT (ρ = 0.27, *p* = 0.00091). Furthermore, Combination Feasibility in the FIT showed marginal positive correlation with Feasibility in the AUT (ρ = 0.16, *p* = 0.056). These results indicate that the FIT assesses an individual’s divergent thinking ability in a manner similar to the AUT.

Second, Fluency and Combination Novelty in the FIT were correlated with other dimensions of the AUT (FIT Fluency vs. AUT Novelty: ρ = 0.31, *p* = 0.00018; FIT Fluency vs. AUT Feasibility: ρ = 0.28, *p* = 0.00062; FIT Combination Novelty versus AUT Fluency: ρ = 0.34, *p* = 2.76e−5). These cross-dimensional correlations between the FIT and the AUT further highlight potential interactions among dimensions, as evidenced both within the FIT (see Fig. 4A) and within the AUT^38^.

Third, Goal Attainment Level in the FIT did not show any significant correlation with any dimension in the AUT: vs. AUT Fluency (ρ = − 0.00029, *p* = 0.997, BF_10_ = 0.07), vs. AUT Novelty (ρ = − 0.005, *p* = 0.95, BF_10_ = 0.07), vs. AUT Feasibility (ρ = − 0.07, *p* = 0.41, BF_10_ = 0.09). This indicates that Goal Attainment Level represents a unique dimension in the FIT, which is specific to solving closed-ended problems.

### The FIT and the AUT reflect different aspects of real-life creativity

Our next step was to evaluate the insights that the FIT provides into an individual’s real-life creativity, and determining whether these insights are distinct from those offered by the AUT (Fig. 4E). For the real-life creativity achievements measured by the CAQ, FIT Combination Novelty was significantly correlated with CAQ-Science (Spearman’s ρ = 0.17, *p* = 0.038), whereas AUT Fluency was significantly correlated with CAQ-Art (ρ = 0.19, *p* = 0.021). For the self-perceived real-life creativity levels measured by the K-DOCS, FIT Fluency was significantly correlated with K-DOCS-Science (ρ = 0.20, *p* = 0.019), whereas AUT Fluency was significantly correlated with K-DOCS-Art (ρ = 0.19, *p* < 0.023). These results suggest that the FIT and the AUT provide complementary information regarding real-life creativity, with FIT and AUT performances associated with creativity primarily in the scientific and artistic domains, respectively. The individual values for significant correlations are further shown in Fig. 4F. Note that there was a reasonable agreement between the CAQ and the K-DOCS. Specifically, CAQ-Science was positively correlated with K-DOCS-Science (ρ = 0.35, *p* = 1.73e−5), and CAQ-Art was positively correlated with K-DOCS-Art (ρ = 0.30, *p* = 0.00027) (see Supplementary Fig. S3).

## Discussion

In the current study, we introduced the FIT as a new closed-ended problem-solving task which not only is framed with real-world problems but also poses minimal demand for domain-specific knowledge. Our results demonstrate that the FIT and the AUT offer complementary assessments for creative problem-solving. To enhance its applicability, we provide 50 questions covering a variety of problems to solve, available in three languages, along with an automated evaluation system that has been validated against human assessments. Furthermore, the design of the questions can be flexibly adapted to specific needs, while the evaluation method should remain applicable.

### Potential strategies to solve FIT

There are several potential approaches for solving the FIT. One approach is to begin with the designated goal and identify an element that could help achieve it; once the element’s potential is recognized, the connection can be refined by incorporating the other element. Alternatively, participants might start by exploring combinations of both elements, selecting the combination that best connects to the goal and then further refining that solution. Depending on the question, or even within a single problem, participants may switch between these strategies. Another intriguing follow-up question in both scenarios is: what is the underlying approach to “combine” two elements? Several possibilities can be considered, including: a ‘relational linking’ process in which combination is based on plausible relationship of the two elements^39^, a ‘property mapping’ process in which combination occurs through transferring the property from one element to another^39^, or a ‘Constraint-guided Conceptual Combination’ process in which combination is guided by evaluation on whether such combination is plausible and effective^40^.

To better understand these problem-solving processes in future studies, we propose an experimental design that incorporates a multi-stage FIT along with post-solution questionnaires. In one condition, participants first generate as many ways as possible to combine the two given elements; then, when presented with the designated goal, they develop FIT solutions based on those combinations. In a second condition, the task is divided into three phases: first, participants generate possible solutions using element 1 alone to achieve the designated goal; next, they generate possible solutions using element 2 alone; and finally, they integrate the insights from the previous phases to develop a comprehensive FIT solution. Comparing performance across these conditions will help us identify which problem-solving approach is more effective for tackling FIT challenges. Furthermore, specific post-solution questions can be designed to retrospectively track the types of conceptual combination strategies.

### The FIT offers a unique measure

The correlation of individual performances of the FIT and the AUT in Fluency, Novelty and Feasibility (marginal) indeed indicates that FIT and AUT may share some underlying process during problem-solving. In particular, both tasks require participants to divergently explore various possibilities of functions or usages of question elements or items. However, the inclusion of a specific goal in FIT poses a clear constraint on where all these possible ideas should converge to. With this additional constraint, the FIT offers additional and complementary information about human creativity in addition to those offered by the AUT.

Another key distinction of the FIT is how it operationalizes the integration of divergent and convergent thinking. Prior studies have measured these processes sequentially: first eliciting multiple ideas through a divergent thinking task, then requiring participants to retrospectively evaluate their ideas as a proxy for convergent thinking (e.g., Runco and Smith^41^). By contrast, the FIT embeds convergence into the generative process itself: participants are required to produce multiple solutions that integrate two given elements toward a defined goal. This constraint demands real-time filtering and synthesis, rather than post hoc selection. In this way, the FIT captures a form of goal-directed generativity that better mirrors the iterative and integrated nature of real-world creative problem-solving. We view this not as a replacement for evaluative-thinking paradigms, but as a complementary tool to study convergent processes under more ecologically relevant constraints.

Furthermore, to achieve the given goal in the FIT, participants apparently need to rely more on their capability to meaningfully and logically integrate two given elements in a creative way, which is evidenced by significant correlation with their real-life creativity in the scientific, rather than artistic, domain. In contrast, in the AUT, there is no clear “goal” to achieve, potentially allowing more space for artistic creativity to play a role. A recent meta-analysis indeed showed an overall stronger link between creative achievement in the artistic and performance domain and divergent thinking task performance (including in the AUT)^27^. Our results also echo previous findings showing higher AUT fluency in professional dancers than lay controls^42^, and a positive correlation between AUT performance and improvisational creative performance in musicians^43^.

### Expandability of FIT evaluation in other language contexts

Using large language models for creativity evaluation additionally improves the usability of the FIT by removing the need for human judges to rate ideas or solutions, which is the typical approach in conventional creativity literature, e.g.^44^. Such an approach also significantly reduces researchers’ burden of worrying about the consistency of rating criteria across human raters^45^. Furthermore, to gain insights about whether this rating approach is also valid in other language contexts of choice, we further tested the GPT-rating performances on FIT solutions in the Traditional Chinese context. The average correlation coefficients between Traditional Chinese GPT ratings and human Elo ratings were 0.55 ± 0.16, 0.42 ± 0.17, and 0.56 ± 0.17 (mean ± standard deviation, n = 50 questions) for Combination Novelty, Combination Feasibility, and Goal Attainment Level, respectively. When comparing to the GPT ratings in the Japanese context (Supplementary Fig. S4), the GPT-rating performances in the Traditional Chinese context were comparable in Combination Novelty (*p* = 0.85, d = 0.03, BF_10_ = 0.16) and Combination Feasibility (*p* = 0.44, d = 0.11, BF_10_ = 0.2), but again slightly lower for Goal Attainment Level (*p* = 0.04, d = 0.31, BF_10_ = 1.3). These results suggest that the GPT-rating approach can be reasonably applied to different language contexts. It is important to note that the current study by no means tries to claim that GPT-4o is the only solution. In a recent study, we demonstrated that different versions of GPT (4, 4 Turbo, and 4 Omni) and Claude 3.5 yielded comparable rating performance^30^. The Elo-rating dataset provided in this study can serve as the ground truth for testing other existing models, such as Llama and Gemini, as well as future models.

### Generalizability of the FIT

The format of the FIT not only reduces the task demand for domain-specific knowledge or skills, but also makes it easier to expand the question sets by randomly pairing two unrelated elements with a goal in real-world contexts. Following this simple rule, one can easily apply the structured prompt in the current study with large language models to generate a custom-made FIT set (see Supplementary Methods). This format further allows users to adjust question contents that match target participants’ age, culture, mental condition or even field of expertise, extending the range of its application to education, health care and even industry. For example, one can flexibly generate different FIT question sets for evaluating creativity outcomes before and after an educational intervention, with a reduced confounding risk from practice effects from using the same set of questions multiple times.

### Acquiring ground truth with Elo-rating

Conventionally, evaluating creativity of answers in tasks like the AUT usually relies on Likert scale judgments from a small group of human raters, who would be trained beforehand to rate creativity with consensual criteria^46^. However, each rater typically needs to rate a huge number of answers, which imposes a heavy cognitive load on raters. Using the Elo-rating approach to establish the ground truth data shows an advantage in creativity evaluation, as compared to conventional Likert-rating approach, with its lowered cognitive load for judges^30,45^. However, there is currently no established standard for the minimum match numbers required to reach steady ratings and the robustness of Elo ratings can be improved with more match numbers^45,47,48^, and some studies suggest that ratings derived from less than 9 matches shall be considered “provisional”^48^. In the current study, the percentages of solutions that were rated less than 9 times were 13.3% for Combination Novelty (1832 solutions), 20.7% for Combination Feasibility (1676 solutions), and 12.4% for Goal Attainment Level (1852 solutions). The GPT-rating performances (correlation coefficient *r*) based on solutions with a match number greater than 9 were 0.54 ± 0.18, 0.39 ± 0.23, and 0.60 ± 0.15 for Combination Novelty, Combination Feasibility, and Goal Attainment Level, respectively (Supplementary Fig. S5A). There was no significant difference between GPT-rating based on all solutions and solutions with a match number greater than 9 for Combination Novelty (*p* = 0.3, Cohen’s d = 0.15, BF_10_ = 0.26), Combination Feasibility (*p* = 0.21, Cohen’s d = 0.18, BF_10_ = 0.33), and Goal Attainment Level (*p* = 0.052, Cohen’s d = − 0.28, BF_10_ = 0.95) (Supplementary Fig. S5B). Therefore, our results are valid according to the suggestions by Neumann et al. (2011).

### Variability in GPT performance across questions

We purposefully designed the GPT-rating approach to be generalizable to any new FIT questions with no training^30^. Even though semantic structures of FIT solutions are more complicated than those of AUT solutions, the GPT performance in the current study was comparable with the automated methods for AUT^30,49,50^. On the other hand, the overall modest correlation (which yields low r^2^ values, indicating low percentage of explained variance) may still raise questions about the validity and feasibility of automatic assessment using large language models (LLMs). In particular, some questions showed weak performance against human ratings (e.g., the Combination Feasibility for Q13, Fig. 2B). From the perspective of data characteristics, this could be due to (1) low variation of ratings across solutions within a question, which reduces the chance to find a meaningful correlation; (2) a low number of solutions within a question, which reduces the power of correlation analysis; or (3) a lower average match number of Elo-ratings which reduces the robustness of the ground truth. Further examination reveals that the standard deviation of GPT ratings show significant or marginal positive correlations with the performance (as measured by correlating with Elo-rating results) across 50 questions in all three dimensions (Supplementary Fig. S6A). On the other hand, no such pattern was observed for the solution counts (Supplementary Fig. S6B) or mean match numbers in Elo-rating (Supplementary Fig. S6C), except for a positive correlation with mean match number for Goal Attainment Level. These results suggest that it might be difficult come up with diversified solutions (i.e., higher standard deviation in ratings) for these questions, due to the inherently limited combinations of elements embedded. Removal or modification of these questions should be considered in future studies.

Another potential explanation for variability in GPT-Elo correlations is the conceptual complexity or familiarity of the elements used in FIT questions. Although we screened items to minimize technical demands, some terms (e.g., “Maglev train technology,” “cryptocurrency”) may still evoke different levels of familiarity across participants. However, our follow-up analysis revealed that several low-performing questions involved relatively simple items (e.g., “weather app,” “motion detector lights”), suggesting that complexity alone may not account for the reduced performance. Future studies should explicitly measure participants’ familiarity with each element and potentially use metrics of semantic distance or conceptual richness to quantify item complexity. Such measures could help refine question selection and scoring reliability. Furthermore, while the FIT emphasizes functional integration over technical knowledge, we acknowledge that the perceived complexity of certain terms could influence how participants construct their solutions. Therefore, future modification of the question sets, including the revision or replacement of poorly performing items, could be helpful to improve the reliability and validity of FIT and the corresponding scoring performance.

From the perspective of the validity of LLMs, the complexity of FIT question design (with two elements and one goal) could increase the challenge to LLMs for providing a valid rating. In the current context, since we did not implement any training on GPT, the results heavily rely on the available mechanisms of GPT without tuning. Apparently, different LLMs are implemented with different underlying algorithms. It is worth to run a thorough comparison among available LLMs (e.g., GPT, Claude, Gemini, Ilama, Mistral Large, Deep Seek) and explore key factors. Furthermore, future automated evaluation methods could also incorporate training with actual data, as demonstrated by Organisciak et al.^50^ for improving their validity.

### Real-world relevance of creativity questionnaires

Using the CAQ and the K-DOCS to evaluate the link between FIT performance and real-world creativity may be insufficient. The CAQ and the K-DOCS are commonly adopted questionnaires in the field – the CAQ tests the link between real-world creativity achievements and creative problem-solving task performance^27,28,51^, intelligence^52–54^, cognitive functions^55–57^, or even dopaminergic polymorphism^58^, while the K-DOCS assesses the link between self-perceived creativity and creative problem-solving task performance^59,60^ or mental disorders^61–64^. However, results from both questionnaires should be interpreted cautiously, with CAQ prone to show a skewed scores (as mentioned in the Method), and K-DOC not covering objective achievements. These concerns could partially account for the modest correlation between FIT/AUT performance and real-world creativity performance. Furthermore, both questionnaires focus more on the consequences of creative ideas, but do not measure the creative potential and ideational behavior. Therefore, future studies could consider using questionnaires such as the Runco Ideational Behavior Scale (RIBS) to capture this additional aspect about the link between FIT performance and creative ideation^65^. On the other hand, the achievement evaluation criteria in the CAQ are relatively strict, which may result in lower scores for younger individuals^31^. To overcome this, the Inventory of Creative Activities and Achievements (ICAA)^66^, which assesses both creative achievements with broadly-defined criteria and engagement in creative activities, and Creative Activity Checklist (CAC)^67,68^, which quantify creative activity participation in both science and art domains, could be added to complement the evaluation from the CAQ.

### Limitations of the current study

We acknowledge that the current results should be considered in light of several methodological limitations. First, in the current study, the task order was fixed for all participants where participants performed FIT before AUT, with questionnaires data collected in-between. It is difficult rule out the possibility that participants’ performance on AUT could be influenced by their experience with FIT or questionnaires, thereby introducing systematic noise on our results related to AUT. Future studies with a counterbalanced task order should be implemented to assess, and reduce, potential ordering effects. Second, the current study was conducted only with a modest sample size of Japanese participants. Considering the multi-faceted nature of creativity, future studies with a more culturally diverse and larger population will be needed to verify if the current results can be replicated.

### Future evaluations on FIT’s psychometric properties

To improve the robustness of FIT and improve its generalizability, it will be necessary to evaluate its psychometric properties with a large and diverse sample size. Specifically, an evaluation of the test–retest reliability and learning effect with different sets of FIT questions will inform how flexible FIT can be used for outcome measurement in the education or creativity enhancement contexts. Although the current results provide a hint about FIT’s criterion validity regarding how well FIT predicts real-world creative achievements, several other types of validity should also be considered to establish the psychometric soundness of FIT. This may include examining its construct validity to test its correlation with other well-established creativity measures or tasks, and its discriminant validity to test whether FIT distinctly measures creative problem-solving rather than general intelligence or other cognitive abilities. Additionally, cross-cultural validation studies should be performed to understand its applicability across different cultural contexts and to identify any potential cultural biases in the assessment. Last but not least, establishing an age-stratified norm for FIT scores will be useful to study the developmental aspects of creativity.

## Conclusion

The current study introduces the FIT as a new problem solving task, which has five unique features: (1) a clear link to real-world creativity; (2) lower demand for domain specific knowledge or skills; (3) a capability to evaluate creativity in the context of solution effectiveness; (4) a structured pipeline to evaluate performances; and (5) high flexibility to adjust or expand the question sets. Our results suggest that the FIT provides an initially promising behavioral paradigm in the closed-ended problem solving category, complementary to the AUT, to study human creativity.

## Supplementary Information

Below is the link to the electronic supplementary material.

Supplementary Material 1

## Data Availability

All data and codes are available at: https://zenodo.org/records/12730520.
