# Supplementary material for: Introducing the fusion innovation test as a new paradigm for studying realworld creative problem solving
Source: Sci Rep. 2025 Dec 23;15:44394. doi: 10.1038/s41598-025-28134-y (PMC12727754; doi:10.1038/s41598-025-28134-y)
Supplement: Supplementary file 1 — Supplementary Material 1 [file 41598_2025_28134_MOESM1_ESM.docx]

Supplementary Materials

# Methods

## GPT prompt-design for the FIT question generation

### FIT Goal-Generation prompts

We first use the following prompts to generate 120 questions with 60 of them targeting on improving personal need in daily life (Self Improvement Goal, SIG) and 60 of them targeting on solving Sustainable Development Goals (SDGs). The generated 120 goals are listed in Supplementary Appendix 1.

Prompts:【

I want to design a creativity test called "Fusion Innovation Test", which aims to evaluate people's real-world creativity. For each question in this test, I will give two "items“ and a specific “goal”. The participant will be given 3.5 minutes to combine two given items to achieve the given goal.

Can you first generate 120 goals with 60 goals related to personal life needs and 60 goals related to sustainable development goals?

The criteria for generating these goals are:

1. The goals should be as diversified as possible and not overlapped.

2. The goals should be specific, and not vague.

3. The goals should be described clearly so that lay people can understand what should be achieved.

Definition of personal life needs: A "personal life need" refers to the individual requirements, desires, and priorities that people have in their day-to-day lives to maintain their well-being, happiness, and functionality. These needs can encompass a wide range of aspects, including physical, emotional, social, and psychological needs. Personal life needs may include basic necessities such as food, shelter, and clothing, as well as emotional needs like love, companionship, and a sense of belonging. They can also involve personal goals, aspirations, and desires, such as achieving a healthy work-life balance, pursuing a hobby, or maintaining physical fitness.

Definition of sustainable development goals: The “Sustainable Development Goals” (SDGs) are a set of global objectives that address a wide range of social, economic, and environmental issues. The key components of the Sustainable Development Goals are:

No Poverty: End poverty in all its forms everywhere.

Zero Hunger: End hunger, achieve food security and improved nutrition, and promote sustainable agriculture.

Good Health and Well-being: Ensure healthy lives and promote well-being for all at all ages.

Quality Education: Ensure inclusive and equitable quality education and promote lifelong learning opportunities for all.

Gender Equality: Achieve gender equality and empower all women and girls.

Clean Water and Sanitation: Ensure availability and sustainable management of water and sanitation for all.

Affordable and Clean Energy: Ensure access to affordable, reliable, sustainable, and modern energy for all.

Decent Work and Economic Growth: Promote sustained, inclusive, and sustainable economic growth, full and productive employment, and decent work for all.

Industry, Innovation, and Infrastructure: Build resilient infrastructure, promote inclusive and sustainable industrialization, and foster innovation.

Reduced Inequality: Reduce inequality within and among countries.

Sustainable Cities and Communities: Make cities and human settlements inclusive, safe, resilient, and sustainable.

Responsible Consumption and Production: Ensure sustainable consumption and production patterns.

Climate Action: Take urgent action to combat climate change and its impacts.

Life Below Water: Conserve and sustainably use the oceans, seas, and marine resources for sustainable development.

Life on Land: Protect, restore, and promote sustainable use of terrestrial ecosystems, sustainably manage forests, combat desertification and halt and reverse land degradation and halt biodiversity loss.

Peace, Justice, and Strong Institutions: Promote peaceful and inclusive societies for sustainable development, provide access to justice for all, and build effective, accountable, and inclusive institutions at all levels.

Partnerships for the Goals: Strengthen the means of implementation and revitalize the global partnership for sustainable development.

】

### FIT Question-Generation prompts

We then randomly paired each goal with 2 items to form a question. The items could be a technology, an App, a machine, an equipment, a tool, a consumer electronic device, an everyday object, an existing service, a transportation vehicle, or a source of data. We also exclude items that were used for the AUT, reducing the interferences of idea generation due to the repetition between the two tasks. Specifically, we used the following prompts to generate 120 FIT questions with variable difficulty levels. The generated 120 questions are listed in Supplementary Appendix 2. We implemented this prompt for 15 goals each time, as more goals tend to lead to missing responses.

prompts:【

I want to design a creativity test called "Fusion Innovation Test", which aims to evaluate people's real-world creativity. For each question in this test, I will give two "items“ and a specific “goal”. The participant will be given 3.5 minutes to combine two given items to achieve the given goal.

For each of the following 15 goals, please pair it with two random “items” to form 15 Fusion Innovation Test questions, randomly with 5 easy ones, 5 moderately difficult ones and 5 difficult ones. The item could be a technology, an App, a machine, an equipment, a tool, a consumer electronic device, an everyday object, an existing service, a transportation vehicle, or a source of data.

The criteria to generate these questions are:

1. The two “items” in each question are not related.

2. The “items” across questions should be as diversified as possible.

3. The “items” should not be repeated across questions or with previous questions.

3. The “items” are clearly and specifically stated and not too technical, so that lay people can understand their functions.

4. The following items cannot be used for any question: Brick, Umbrella, Ketchup, Rubber band, Newspaper, Paper cup, PET bottle, Slippers, Salt, Wood chopsticks, Drinking straw, Plastic bag, Handkerchief, Needle, T-shirt, Shoelace, Metal key, Candle, Toilet paper, Tape, Magnifier, Ball pen, Marble, CD, Carton, Cork, Credit card, Flashlight, Toothbrush, Coin, Facial mask, Baseball cap, rubber gloves, rubber eraser, tennis racket, clothes hanger, backpack, post-it notes, belt, hose, coffee filter, tennis ball, safety pins, clothe drying pole, pillow, bowl, ring, bottle opener, comb, clothe pins.

Please also provide 1 valid solution that achieves the given goal for each question.

Goals:

……….(list of 15 goals from the list)

】

### Question trimming

Next, we invited 10 evaluators to subjectively evaluate each question with the following 3 criteria:1. comprehension of both items (Yes or No), 2. comprehension of the given goal (Yes or No), and 3. the subjective difficulty to come up with a valid solution (Easy, Neutral or Difficult). Based on the results, we removed questions with a comprehension rate lower than 80% (i.e., any question with less than 8 evaluators understanding the items or the goal). In the end, we have 25 questions for the SIG condition and 25 questions for the SDG condition. The 50 questions were divided into 5 sets of questions with each set containing 5 questions for the SIG condition and 5 questions for the SDG condition. Furthermore, we avoid having questions that share similar items within the same set, so as to prevent participants from recycling thoughts from other questions. The contents of the 50 questions are provided in Table 2, Table S1, and Table S2.

## GPT prompts for translating FIT solutions into English and Traditional Chinese

All FIT solutions were translated into English or Traditional Chinese using the following prompt with GPT-4o. The {} indicates variables that need to be inserted.

Prompts:【

Goal: We ask participants to provide solutions for a Fusion Innovation Task (FIT). People are required to come up with creative solutions that combine the 2 provided elements: "{Element_1}" and "{Element_2}" to achieve the goal: "{Goal}." In a solution, the 2 given elements must be used, but they were allowed to add additional elements into their solutions whenever needed. All the participants provide solutions in Japanese. I want you to provide accurate and readable translations from Japanese to {Target_Language}. Please focus on accuracy and clarity given the context above. Translate the text faithfully to the original meaning.

Instructions: Evaluate each solution in the order provided, and answer in one JSON. The JSON should be a list (length of {chunk_size}) of dictionaries with a key: "Translation" that store your translated text. Do translate each of the {N_Answers} solutions below individually, even if there are repetitions.

Japanese responses:

{input of FIT solutions}”】

## GPT prompts for generating sanity check solution pairs

For Combination Novelty:

Prompts:【Here is a question for Fusion Innovation Test, please provide two example solutions, with one to be extremely Novel (unique, surprising) but NOT feasible (practical or doable in real- life setting), and the other solution be extremely NOT Novel (unique, surprising) but feasible (practical or doable in real-life setting). The solutions should be written in condensed sentences (less than 2). The solution should very briefly mention how does it achieve the goal. Element: 1. [xxx] 2. [xxx] Goal: [xxx].】

For Combination Feasibility:

Prompts:【Here is a question for Fusion Innovation Test, please provide two example solutions, with one to be extremely feasible (practical or doable in real-life setting), and the other solution be extremely NOT feasible (practical or doable in real-life setting). The solutions should be written in condensed sentences (less than 2). The solution should very briefly mention how does it achieve the goal. The solution should very briefly mention how does it achieve the goal. Element: 1. [xxx] 2. [xxx] Goal: [xxx].】

For Goal Attainment Level

For high Goal Attainment Level, we use high feasible solutions generated by above mentioned GPT prompts. In contrast, for low Goal Attainment Level, we pick a random solution generated by GPT prompts by a totally different question, thus creating an obvious mismatch between the given goal and the solution.

All the sanity check solution pairs are provided in the data sharing link that corresponds to the current study.

# Supplementary Tables

Table S1 The Japanese version of FIT question

| **MIT questions** | **Element 1** | **Element 2** | **Goal** |
| --- | --- | --- | --- |
| *Self Improvement Goal (SIG)* |  |  |  |
|  | デジタルカメラ | 塗り絵本 | 個人の創造性と表現を培う |
|  | バーチャルリアリティ（VR）ヘッドセット | ペットの魚 | 孤独感や孤立感を和らげる |
|  | 卓上ランプ | 浄水フィルター | 定期的な水分摂取を促進する |
|  | 冷蔵庫 | アレルギー警告アプリ | 食事制限の管理を支援する |
|  | 南京錠 | ウェブブラウザー | 個人のデジタルプライバシーを保護する |
|  | ショッピングカート | バーコードスキャナー | 食料品の買い物を簡易化する |
|  | ボイスレコーダー | 言語学習アプリ | 個人のコミュニケーションスキルを高める |
|  | ストレスリリースボール | メディテーションアプリ | 困難な時期に耐え対処する能力を強化する |
|  | YouTube | パズル | 新しいスキルの取得を支援する |
|  | エレベーター | 電子図書館 | 書籍や教材へのアクセスを容易にする |
|  | 貯金箱 | スプレッドシートソフトウェア | 個人の予算・貯蓄管理を簡易化する |
|  | ベビーモニター | 子育てブログ | 効果的な子育てのためのツールを提供する |
|  | デジタル財布 | レビューのプラットフォーム | 安全なオンラインショッピング体験を保証する |
|  | ミキサー | 健康・栄養データベース | 免疫システムの健康を強化する |
|  | GPSデバイス | 衣類クローゼット | 衣類の選択と身だしなみを整える日課を改善する |
|  | ウェブカメラ | 動作感知ライト | ホームセキュリティーを向上させる |
|  | ハンモック | 瞑想アプリ | 精神的なリラクゼーションを促進する |
|  | 音楽ストリーミングアプリ（例、Spotify） | 鉢植え | 心理的・精神的なウェルビーイング（well being）を改善する |
|  | 書類整理棚 | スマートフォンのリマインダーアプリ | パーソナルスペースの管理を改善する |
|  | 対話型クイズアプリ | 電子レンジ | 正規教育外での学びの機会を増やす |
|  | 掃除機 | カレンダーアプリ | 家事をより効率的にする |
|  | 歩行杖 | ビデオ電話デバイス | 高齢の家族のケアを支援する |
|  | スマートフォン | 医学ジャーナル | 質の高いヘルスケア情報にアクセスできるようにする |
|  | スマートウォッチ | 唐辛子スプレー | 個人の安全を向上させる |
|  | 公園のベンチ | ソーシャルネットワーキングサイト | 積極的なコミュニティーの関与を促進する |
| *Sustainable Development Goal (SDG)* |  |  |  |
|  | 電気自動車 | 旅のレビューのウェブサイト | 環境に配慮した旅を促進する |
|  | モバイルアラートアプリ | コミュニティーラジオ局 | 災害への備えを改善する |
|  | 駐車場 | セラミックのフィルター | 効果的な貯水のための雨水集水システムを創出する |
|  | 輸送用コンテナ | 屋上緑化システム | 手ごろな値段で入手可能であり、 環境への影響を最小限にした住居を考案する |
|  | 暗号通貨 | 求人ポータルサイト | 同一労働同一賃金を奨励する |
|  | アニメーション制作 | 気象台データ | 気候変動についての教育を促進する |
|  | バーチャルリアリティー（VR）会議 | 大学の研究室 | 持続可能な技術を共有するための連携を促進する |
|  | コーヒーメーカー | 竹炭 | 人間用の飲料水を得るための効果的な水質浄化法を開発する |
|  | みつろう | アイスキャンデーの棒 | 環境に配慮した革新的なパッケージングのアイディアを提案する |
|  | 3D プリンター | ローカル職人の工芸品 | コミュニティーにおける環境に配慮した製品生産を促進する |
|  | デジタル広告版 | ドローン | 国土保全についての市民意識を高める |
|  | バーチャルリアリティー（VR ）ゲーム | 学校のカリキュラム | SDGs（Sustainable Development Goals：持続可能な開発目標）に関連するプロジェクトへ関わることを若者に促す |
|  | 天気アプリ | カメラ付きドアベル | 自然災害を早期に警告するツールを考案する |
|  | 磁気浮上式鉄道技術 | カーシェリングアプリ | 効率的な公共交通システムを開発する |
|  | 藻類 | 地下鉄システム | 二酸化炭素を回収・貯留する方法を考案する |
|  | 野生動物のドキュメンタリー | 拡張現実（AR）グラス | 生物多様性の保全を促進する |
|  | 図書館システム | 整備済ラップトップ | 貧困コミュニティーにおいてテクノロジーへのアクセスを促進する |
|  | スポーツジム設備 | ソーラーパネル | クリーンエネルギーを生産するためのシステムを考案する |
|  | 自動販売機 | リサイクリングアプリ | 電子廃棄物の持続可能な解決策を考案する |
|  | フィットネストラッカー | ローカルの農産物直売所 | 貧困コミュニティーにおいて健康に関連するプロジェクトを促進する |
|  | 屋上 | 水耕庭園システム | ローカルでの食糧生産を高める |
|  | 空気清浄器 | 公共バス | 大気清浄イニシアチブを通して公衆衛生を向上させる |
|  | 国際映画祭 | 環境に配慮した技術 | SDGs（Sustainable Development Goals：持続可能な開発目標）に向けた国際パートナーシップを促進する |
|  | 竹繊維 | QR コード | 環境により配慮した衣類を促進する |
|  | 電子文書リーダー | ソーラーランタン | 貧困コミュニティーにおけるデジタルリテラシーを向上させる |

Table S2 The Traditional Chinese version of FIT

| **MIT questions** | **Element 1** | **Element 2** | **Goal** |
| --- | --- | --- | --- |
| *Self Improvement Goal (SIG)* |  |  |  |
|  | 數位相機 | 著色本 | 培養個人的創造力和表達力 |
|  | 虛擬實境（VR）頭戴裝置 | 寵物魚 | 減少寂寞和孤立感 |
|  | 桌燈 | 濾水器 | 鼓勵規律飲水 |
|  | 冰箱 | 過敏警示應用程式 | 協助飲食禁忌的管理 |
|  | 掛鎖 | 網頁瀏覽器 | 保護個人數位隱私 |
|  | 購物車 | 條碼掃描器 | 簡化雜貨購物 |
|  | 錄音工具 | 語言學習應用程式 | 增進個人溝通技能 |
|  | 壓力球 (或擠壓球) | 冥想應用程式 | 強化個人對於艱困處境的承受和管理能力 |
|  | YouTube | 各式謎題 | 輔助個人學習一個新技能 |
|  | 電梯 | 電子圖書館 | 建立更便利的方式來取得書籍或學習資料 |
|  | 存錢筒 | 試算表軟體 | 簡化個人預算和儲蓄 |
|  | 嬰兒監視器 | 育兒部落格 | 提供有效的育兒方法、策略或工具 |
|  | 數位錢包 | 評論平台 | 確保安全的網路購物體驗 |
|  | 果汁機 | 關於健康和營養的資料庫 | 強化個人免疫系統的健康 |
|  | GPS裝置 | 衣櫥 | 改善個人的時尚選擇和日常個人儀容的整理 |
|  | 視訊鏡頭 | 動作感應燈 | 提升住家安全 |
|  | 吊床 | 冥想應用程式 | 促進心情放鬆 |
|  | 音樂串流應用程式（如Spotify） | 盆栽植物 | 改善心理安適 |
|  | 文件櫃 | 智慧型手機中的提醒應用程式 | 改善個人空間的整理與規劃 |
|  | 互動式的問答應用程式 | 微波爐 | 增加正式教育以外的學習機會 |
|  | 吸塵器 | 日曆應用程式 | 提高家務處理的效率 |
|  | 拐杖 | 視訊通話裝置 | 提供對於年紀大的家人的照護輔助 |
|  | 智慧型手機 | 醫學期刊 | 建立獲得高品質醫療資訊的管道 |
|  | 智慧型手錶 | 防狼噴霧 | 強化個人安全 |
|  | 公共公園的長椅 | 社交網站 | 鼓勵積極的社區參與 |
| *Sustainable Development Goal (SDG)* |  |  |  |
|  | 電動車 | 旅遊評論網站 | 推廣對環境友善的旅行 |
|  | 手機警示通知程式 | 社區廣播電台 | 改善對災害的準備 |
|  | 停車場 | 陶瓷濾芯 | 創建一個雨水收集系統來有效的儲水 |
|  | 運輸用貨櫃 | 屋頂綠化系統 | 設計多數人可負擔的平價環保住宅（對環境產生極低限度的影響） |
|  | 加密貨幣 | 求職平台 | 鼓勵同工同酬 |
|  | 動畫影集（動畫製作） | 氣象站數據 | 促進關於氣候變遷的教育 |
|  | 虛擬實境會議 | 大學研究實驗室 | 促進合作來共享永續發展的技術 |
|  | 咖啡機 | 竹炭 | 開發對於飲用水更有效的淨水方法 |
|  | 蜂蠟 | 冰棒棍 | 提出創新的環保包裝設計 |
|  | 3D列印機 | 在地的職人手工藝品 | 鼓勵在地社區產品的環保製造 |
|  | 數位告示牌 | 無人機 | 增進土地保存的公眾意識 |
|  | 虛擬實境（VR）遊戲 | 學校課程 | 鼓勵青少年參與關於永續發展目標 (SDG, Sustainable Development Goals) 的計畫 |
|  | 氣象預報的應用程式 | 有監控攝影功能的門鈴 | 設計天然災害的早期預警系統 |
|  | 磁浮列車技術 | 共乘應用程式 | 發展高效率的大眾運輸系統 |
|  | 藻類 | 地下鐵系統 | 設計碳捕獲與儲存的方法 |
|  | 野生動物紀錄片 | 擴增實境（AR）眼鏡 | 鼓勵生態多樣性的保存 |
|  | 圖書館系統 | 整修過的筆記型電腦 | 於貧困社區促進使用科技的機會 |
|  | 健身房設備 | 太陽能板 | 設計乾淨能源的發電系統 |
|  | 自動販賣機 | 回收應用程式 | 設計電子廢棄物的永續解決方案 |
|  | 健身追蹤器 | 在地農產品市場 | 在貧困社區推廣健康相關的方案 |
|  | 屋頂 | 水培系統（無土種植系統） | 增強在地的食品生產 |
|  | 空氣淨化器 | 公共巴士 | 設計一個乾淨空氣的計劃來增進大眾健康 |
|  | 國際電影節 | 綠色技術（環保技術） | 鼓勵永續發展目標(SDG, Sustainable Development Goals) 的國際合作 |
|  | 竹纖維 | 二維碼 (QR code) | 鼓勵永續時尚 (推廣對於環境友善的服飾) |
|  | 電子閱讀器 | 太陽能燈 | 提升貧困社區對於數位文化的認識 |

Table S3 AUT questions in English, Japanese and Traditional Chinese

| AUT sets | Items | | |
| --- | --- | --- | --- |
|  | English | Japanese | Traditional Chinese |
| 1 | brick | レンガ | 磚頭 |
| 1 | newspaper | 新聞紙 | 新聞報紙 |
| 1 | magnifier | 虫眼鏡 | 放大鏡 |
| 1 | cd | CD | CD |
| 1 | toothbrush | 歯ブラシ | 牙刷 |
| 1 | coin | コイン | 硬幣 |
| 1 | rubber gloves | ゴム手袋 | 橡膠手套 |
| 1 | tennis racket | テニスラケット | 網球拍 |
| 1 | belt | ベルト | 皮帶 |
| 1 | pillow | 枕 | 枕頭 |
| 2 | umbrella | 傘 | 傘 |
| 2 | ketchup | ケチャップ | 番茄醬 |
| 2 | rubber band | 輪ゴム | 橡皮筋 |
| 2 | plastic bottle | ペットボトル | 寶特瓶 |
| 2 | metal key | 金属製の鍵 | 金屬鑰匙 |
| 2 | cork | コルク | 軟木塞 |
| 2 | flashlight | 懐中電灯 | 手電筒 |
| 2 | baseball cap | 野球帽 | 棒球帽 |
| 2 | bowl | お椀 | 碗 |
| 2 | comb | くし | 梳子 |
| 3 | paper cup | 紙コップ | 紙杯 |
| 3 | slippers | スリッパ | 拖鞋 |
| 3 | shoelace | 靴ひも | 鞋帶 |
| 3 | candle | ろうそく | 蠟燭 |
| 3 | carton | 段ボール箱 | 紙箱 |
| 3 | facial mask | マスク | 口罩 |
| 3 | rubber eraser | 消しゴム | 橡皮擦 |
| 3 | coffee filter | コーヒーフィルター | 咖啡濾紙 |
| 3 | bottle opener | 栓抜き | 開瓶器 |
| 3 | clothespins | 洗濯ばさみ | 曬衣夾 |
| 4 | salt | 塩 | 鹽 |
| 4 | drinking straw | ストロー | 吸管 |
| 4 | needle | 裁縫針 | 縫衣針 |
| 4 | T-shirt | Tシャツ | T恤衫 |
| 4 | credit card | クレジットカード | 信用卡 |
| 4 | backpack | バックパック（リュックサック） | 背包 |
| 4 | hose | ホース | 水管 |
| 4 | tennis ball | テニスボール | 網球 |
| 4 | safety pins | 安全ピン | 安全別針 |
| 4 | ring | 指輪 | 戒指 |
| 5 | wood chopsticks | 木の箸 | 木筷 |
| 5 | plastic bag | ビニール袋 | 塑膠袋 |
| 5 | handkerchief | ハンカチ | 手帕 |
| 5 | toilet paper | トイレットペーパー | 廁所衛生紙 |
| 5 | Tape | テープ | 膠帶 |
| 5 | ballpoint pen | ボールペン | 原子筆 |
| 5 | marble | ビー玉 | 彈珠 |
| 5 | clothes hanger | ハンガー | 衣架 |
| 5 | post-it notes | ポストイット付箋 | 便利貼 |
| 5 | clothes drying pole | 物干しざお | 曬衣桿 |

Note. The current AUT intended to include items that people may encounter in everyday life, therefore, items like salt or ketchup were included, even though they are not human-made artifacts as in conventional AUT.

Supplementary Figures


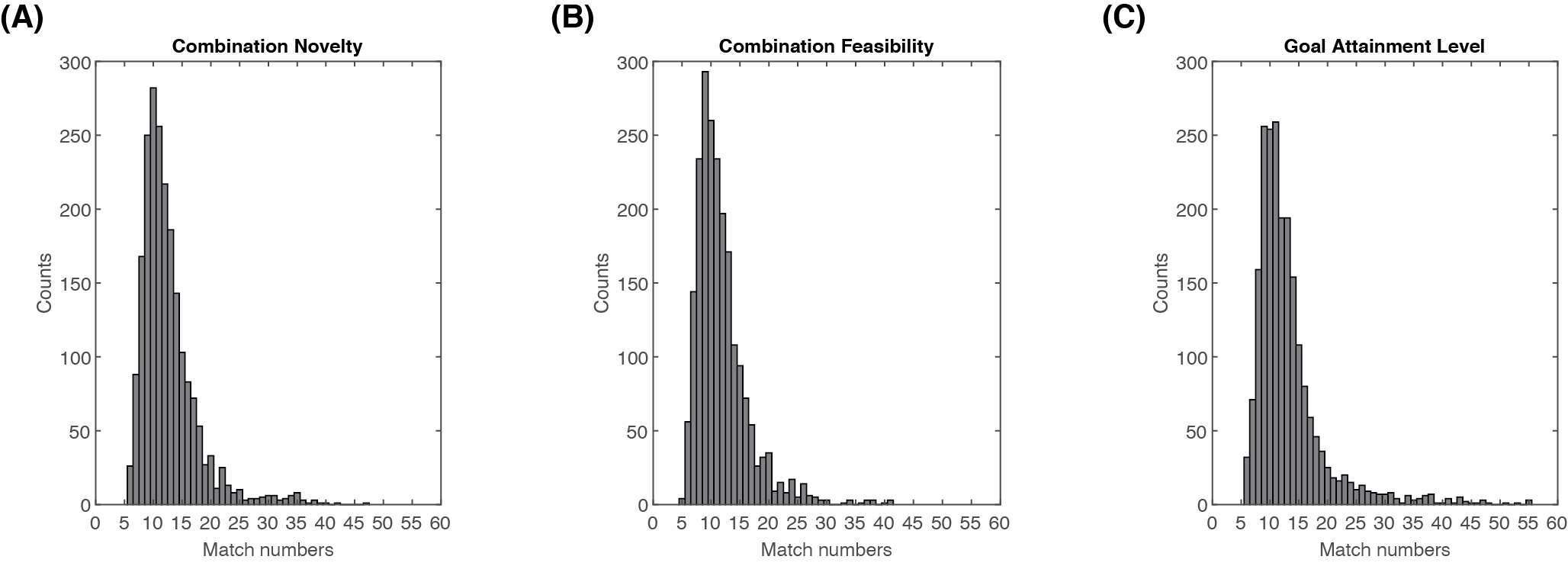


**Figure S1. Histograms of match counts for all solutions in FIT. (A)** Combination Novelty. **(B)** Combination Feasibility. **(C)** Goal Attainment Level. The x-axis represents the match numbers and the y-axis represents the counts of solutions.


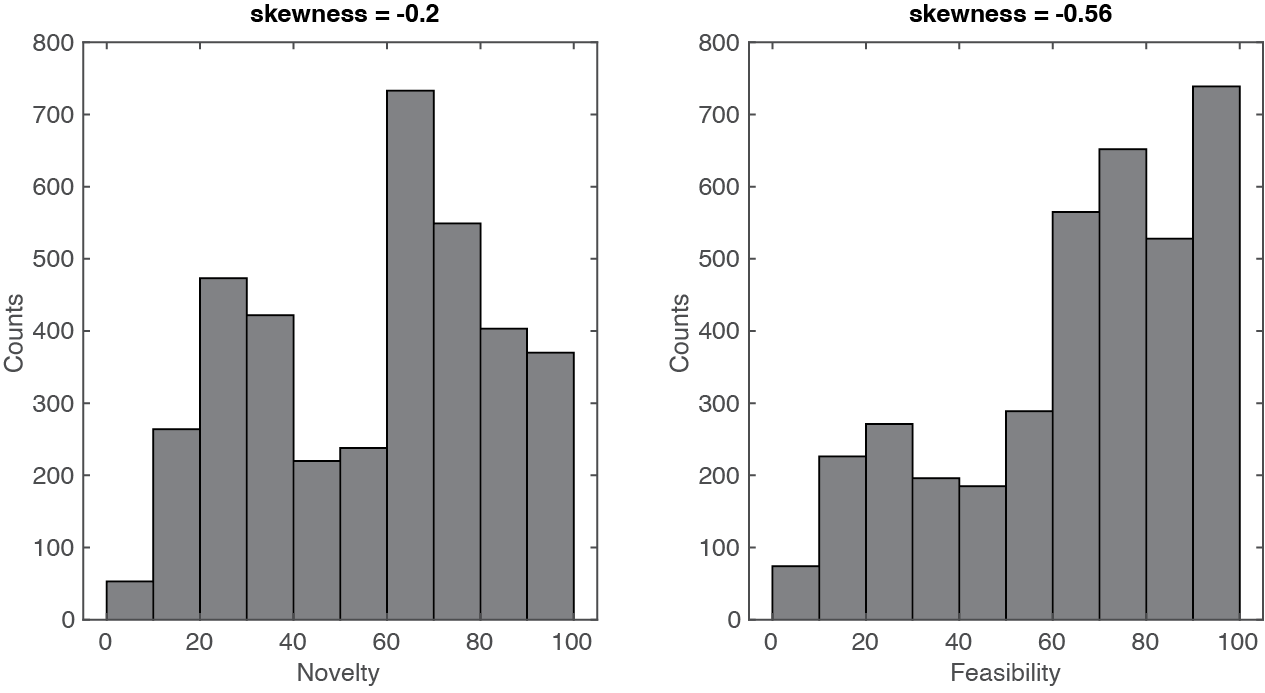


**Figure S2. Distributions of each measurement dimension within the AUT.** The histograms of score distribution for Novelty and Feasibility. The corresponding skewness values are provided at the top of each histogram.


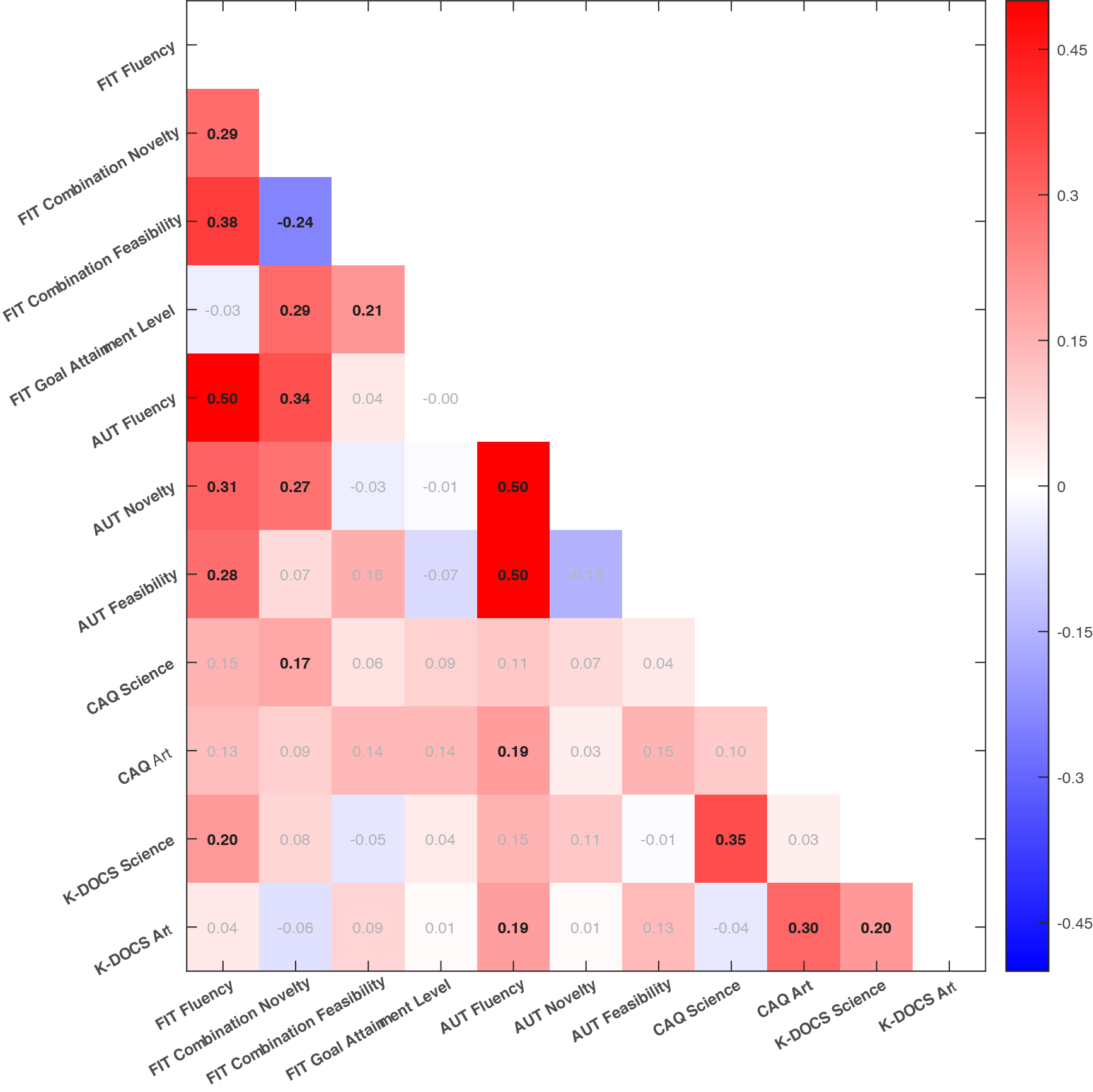


**Figure S3. The correlation matrix between measurement dimensions of FIT, AUT, CAQ, and K-DOCS.** The complete correlation matrix between all measurement dimensions of FIT, AUT, CAQ and K-DOCS. The colorbar indicates the strength of correlation as calculated by Spearman’s ρ, with red representing positive correlation and blue representing negative correlation. Significant correlations are highlighted in black and bold texts.


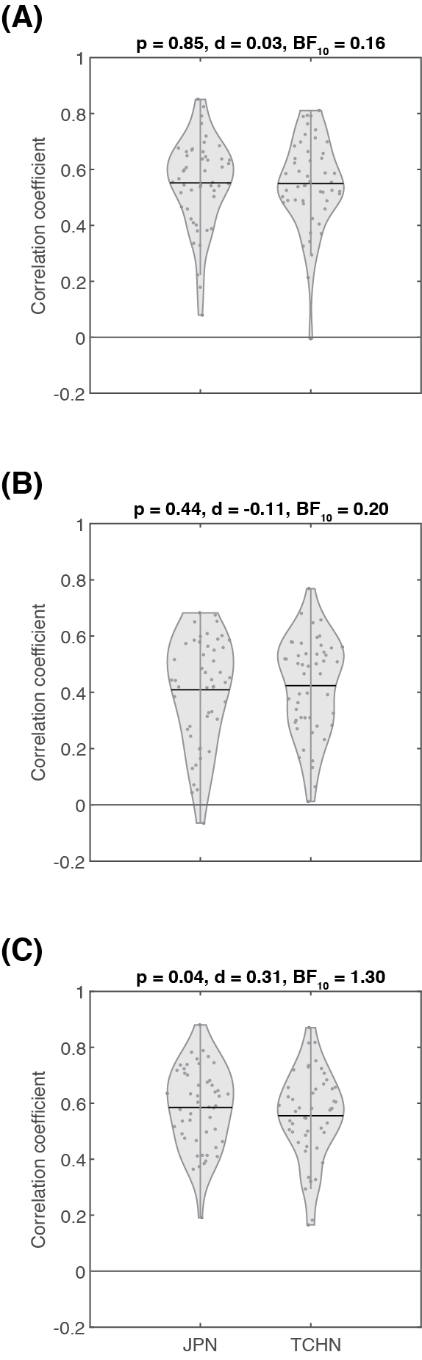


**Figure S4. The GPT-rating performances in the Traditional Chinese context.** The violine plots of the distribution of 50 weighted rank correlation coefficients between Elo-rating and GPT-rating under Japanese context (JPN) and Traditional Chinese context (TCHN) for Combination Novelty **(A)**, Combination Feasibility **(B)** and Goal Attainment Level **(C)**. The black solid lines in each plot represent the means. The results of statistical tests between two language contexts are provided on the top of each violine plot. Note. d = Cohen’s d; BF_10_ = Bayes Factor.


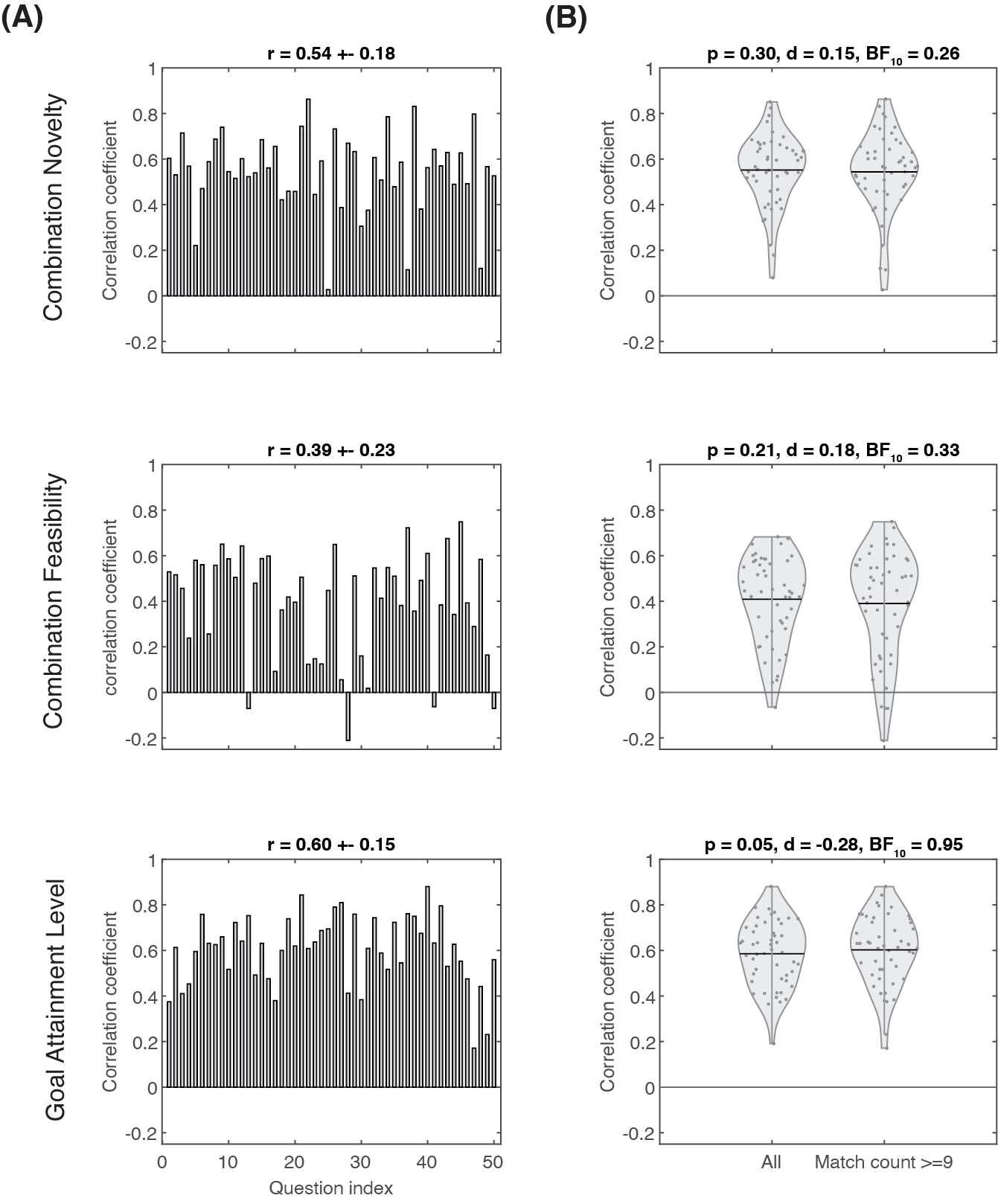


**Figure S5. Summary of GPT-rating performances on all FIT solutions vs. solutions with a match number >=9.** **(A)** The bar plots show *r* values for FIT solutions with an Elo-rating match umber >=9 across all 50 questions for Combination Novelty (top), Combination Feasibility (middle), and Goal Attainment Level (bottom), under the Japanese context. The corresponding mean and standard deviation of the 50 weighted correlation coefficients *r* are provided on the top of each bar graph. The black bars indicate the values for Question No. 7 as shown in panel A. **(B)** The violine plots of the distribution of 50 *r* values under the Japanese contexts with all solutions (All) vs. solutions with match number >=9 (match count >=9) for Combination Novelty (top), Combination Feasibility (middle), and Goal Attainment Level (bottom). The black solid lines in each plot represent the means. The results of statistical tests between two language contexts are provided on the top of each violine plot. The corresponding correlation coefficient *r* (mean ± standard deviation) is shown. Note. d = Cohen’s d; BF_10_ = Bayes Factor.


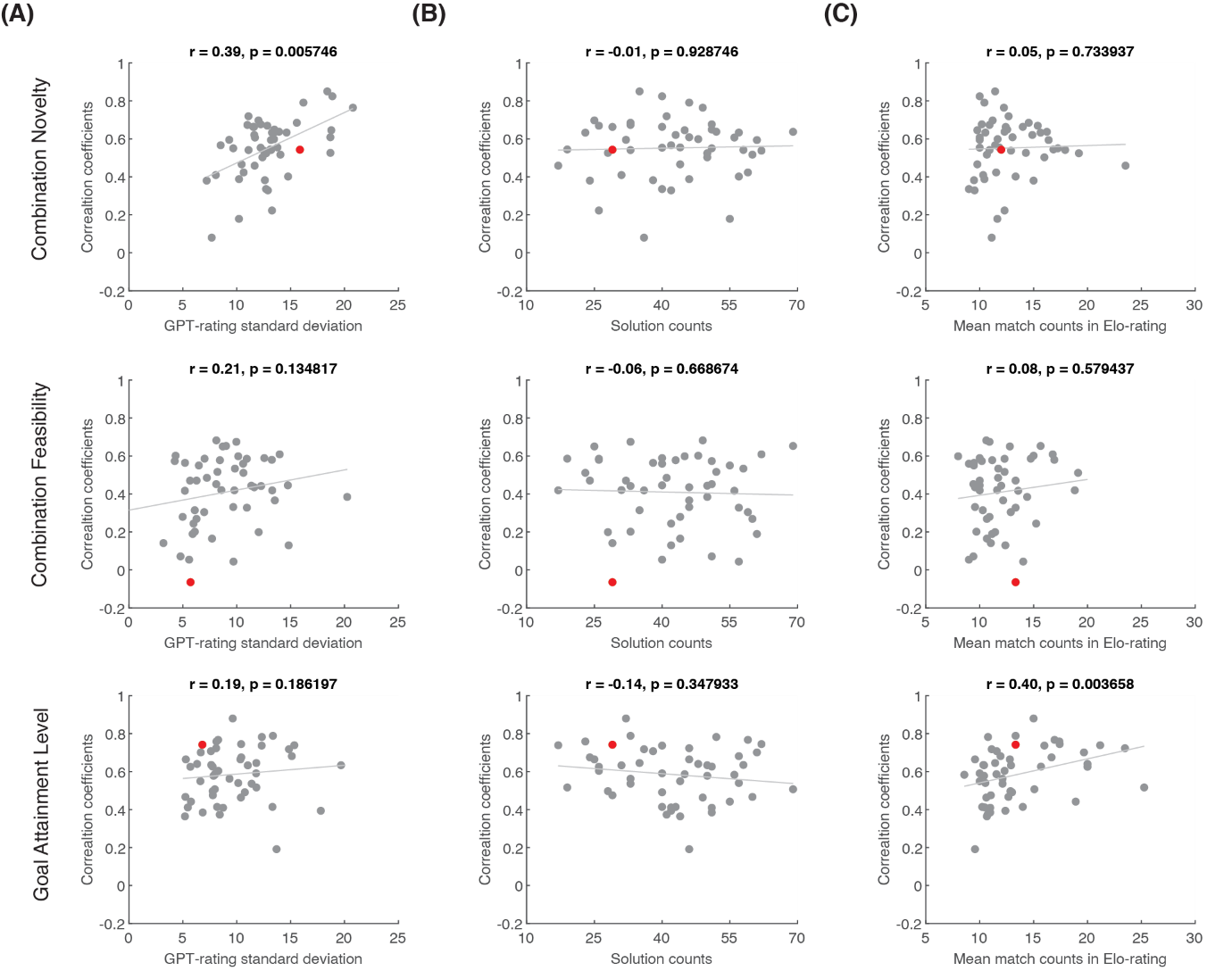


**Figure S6. Summary of GPT-rating performances on all FIT solutions against standard deviation of GPT ratings, solution counts and mean match counts in Elo-rating cross questions.** The scatter plot of the GPT-rating performances (y-axis) against GPT-rating standard deviation (x-axis) **(A)**, solution counts (x-axis) **(B)**, and mean match numbers in Elo-rating (x-axis) **(C)** across 50 questions for Combination Novelty (top), Combination Feasibility (middle), and Goal Attainment Level (bottom). The corresponding correlation coefficients *r* (Spearman) and p-values are provided on the top of each graph. Linear regression lines are shown for visualization purposes. The red dots indicate an example of Question No13, which shows a low performance in Combination Feasibility.

**Supplementary Appendix 1**

**Goals generated by GPT4**

*Personal Life Needs Goals (60):*

1. Improve mental well-being and reduce stress.
2. Create a system for efficient meal planning.
3. Increase daily physical activity.
4. Foster deeper personal connections and friendships.
5. Enhance personal safety in public spaces.
6. Streamline household chores.
7. Boost self-confidence and self-esteem.
8. Enhance learning opportunities outside of formal education.
9. Find methods to ensure adequate, restful sleep.
10. Strengthen financial management skills.
11. Facilitate efficient personal transportation.
12. Reduce feelings of loneliness and isolation.
13. Assist in pursuing a new hobby or skill.
14. Facilitate better management of personal time.
15. Help create daily routines that ensure productivity.
16. Enable access to quality healthcare information.
17. Encourage regular health check-ups.
18. Enhance home security.
19. Provide tools for effective parenting.
20. Ensure personal digital privacy.
21. Improve indoor air quality.
22. Facilitate better posture during work or study.
23. Boost motivation for personal goals.
24. Enable easier access to books or learning materials.
25. Aid in setting and tracking personal goals.
26. Foster personal creativity and expression.
27. Encourage regular hydration.
28. Simplify grocery shopping.
29. Promote relaxation and mental breaks.
30. Facilitate household repair and maintenance.
31. Encourage safe online interactions.
32. Assist in managing dietary restrictions.
33. Enhance personal wardrobe and grooming.
34. Encourage positive community engagement.
35. Improve personal space organization.
36. Enhance personal digital literacy.
37. Encourage environmental conservation at an individual level.
38. Enhance pet care.
39. Boost resilience and coping mechanisms.
40. Improve personal energy levels.
41. Encourage regular dental check-ups.
42. Simplify personal budgeting and saving.
43. Ensure a safe online shopping experience.
44. Enhance personal communication skills.
45. Aid in the care of elderly family members.
46. Strengthen personal relationship bonds.
47. Improve home energy efficiency.
48. Foster a better work-life balance.
49. Encourage continuous self-growth.
50. Enhance indoor lighting.
51. Improve local travel experiences.
52. Enhance personal spiritual or meditation practices.
53. Foster intergenerational bonding within families.
54. Strengthen immune system health.
55. Encourage sustainable personal shopping habits.
56. Improve the comfort of personal living spaces.
57. Aid in personal crisis management.
58. Encourage self-reflection and mindfulness.
59. Simplify home gardening and plant care.
60. Enhance personal entertainment experiences.

*Sustainable Development Goals (60):*

1. Develop methods to upcycle waste.
2. Design systems for clean energy generation in urban settings.
3. Improve education access in remote areas.
4. Promote sustainable fishing practices.
5. Foster gender equality in workplaces.
6. Enhance potable water purification methods.
7. Design affordable housing with minimal environmental impact.
8. Foster sustainable farming practices.
9. Improve public health in low-income areas.
10. Encourage sustainable tourism.
11. Promote community-based renewable energy projects.
12. Develop efficient public transportation systems.
13. Encourage reforestation efforts.
14. Design tools for monitoring air quality.
15. Create awareness about responsible consumption.
16. Promote gender equality in political representation.
17. Develop efficient waste management systems.
18. Foster innovation in sustainable packaging.
19. Promote equal access to technology in underserved communities.
20. Design water conservation systems for agriculture.
21. Improve disaster preparedness in vulnerable communities.
22. Foster education on climate change.
23. Create sustainable urban green spaces.
24. Design products using sustainable materials.
25. Encourage biodiversity conservation.
26. Promote peacebuilding in conflict zones.
27. Improve sanitation facilities in underserved areas.
28. Foster community-based health initiatives.
29. Encourage sustainable fashion.
30. Strengthen institutions for corruption prevention.
31. Design products to reduce carbon footprint.
32. Encourage youth involvement in sustainability projects.
33. Enhance public awareness on marine conservation.
34. Foster skills training for sustainable job opportunities.
35. Design affordable, sustainable energy storage solutions.
36. Encourage partnerships for sustainability between countries.
37. Enhance waste reduction in food production.
38. Develop tools for monitoring water quality.
39. Promote sustainable livestock farming.
40. Encourage equal pay for equal work.
41. Foster community resilience to climate change.
42. Promote conservation of freshwater resources.
43. Encourage local sustainable product manufacturing.
44. Foster inclusive education for differently-abled individuals.
45. Design infrastructure for efficient rainwater harvesting.
46. Enhance digital literacy in underserved communities.
47. Design tools for early warning systems for natural disasters.
48. Promote sustainable urban transportation.
49. Enhance local food production in urban areas.
50. Design sustainable solutions for electronic waste.
51. Foster child rights and child protection measures.
52. Promote eco-friendly travel.
53. Enhance public awareness on land conservation.
54. Encourage fair trade practices.
55. Develop solutions to combat desertification.
56. Design methods for carbon capture and storage.
57. Foster economic growth using sustainable practices.
58. Enhance public health through clean air initiatives.
59. Foster community-based wildlife conservation.
60. Encourage partnerships for technology transfer in sustainability.

**Supplementary Appendix 2**

**FIT questions generated by GPT4**

**Easy Questions:**

1. **Goal**: Improve mental well-being and reduce stress.
   - **Items**: Music streaming app (like Spotify) & Potted Plant
   - **Solution**: Create an app that plays calming music and sounds when it detects you're near your potted plants, combining the benefits of soothing audio with the relaxation benefits of nature.
2. **Goal**: Foster deeper personal connections and friendships.
   - **Items**: Smartphone & Board Game
   - **Solution**: Develop a board game app that requires in-person interaction and challenges, using the smartphone as the board and leveraging its sensors and features for gameplay.
3. **Goal**: Streamline household chores (Make household tasks more efficient).
   - **Items**: Vacuum cleaner & Calendar app
   - **Solution**: A smart vacuum cleaner that connects to your calendar app. It schedules cleaning sessions during times you're out or busy, based on your calendar events.
4. **Goal**: Assist in pursuing a new hobby or skill.
   - **Items**: YouTube & Puzzle
   - **Solution**: Develop an interactive YouTube channel or platform where learning a skill is presented in puzzle challenges, making the process engaging and progressive.
5. **Goal**: Find methods to ensure adequate, restful sleep.
   - **Items**: Weighted blanket & Sound machine
   - **Solution**: A weighted blanket with an integrated sound machine that produces white noise, simulating a cozy environment conducive for restful sleep.

**Moderate Questions:**

1. **Goal**: Enhance personal safety in public spaces.
   - **Items**: Smartwatch & Pepper spray
   - **Solution**: A smartwatch feature that discreetly alerts authorities and shares your location when a certain gesture is made, combined with a compact pepper spray release for immediate defense.
2. **Goal**: Create a system for efficient meal planning.
   - **Items**: Refrigerator & Pinterest
   - **Solution**: A smart refrigerator that scans its contents, then suggests recipes from Pinterest based on the ingredients available, helping in efficient meal planning.
3. **Goal**: Boost self-confidence and self-esteem.
   - **Items**: Mirror & Augmented Reality (AR) glasses
   - **Solution**: AR glasses that work with a mirror to overlay positive affirmations or virtual makeovers, boosting the user's self-confidence during their morning routine.
4. **Goal**: Reduce feelings of loneliness and isolation.
   - **Items**: Virtual Reality (VR) headset & Pet fish
   - **Solution**: A VR platform where the user interacts in a virtual space that feels like an underwater world, with their pet fish as a companion or guide, providing a sense of connection.
5. **Goal**: Facilitate better management of personal time.

- **Items**: Alarm clock & Fitness tracker
- **Solution**: An alarm clock that syncs with a fitness tracker, analyzing when you're in light sleep to wake you up at optimal times and setting reminders based on your activity levels.

**Difficult Questions:**

1. **Goal**: Increase daily physical activity.

- **Items**: E-book reader & Staircase
- **Solution**: An e-book reader built into staircases, where pages are turned or content continues only as you climb, encouraging users to keep moving.

1. **Goal**: Enhance learning opportunities outside of formal education.

- **Items**: Microwave oven & Interactive Quiz App
- **Solution**: A microwave that plays short educational quizzes during cooking times, turning waiting periods into learning opportunities.

1. **Goal**: Strengthen financial management skills.

- **Items**: Piggy bank & GPS system
- **Solution**: A piggy bank linked to a GPS system that tracks and rewards you for visiting budget-friendly or discount locations, encouraging money-saving behaviors.

1. **Goal**: Facilitate efficient personal transportation.

- **Items**: Bicycle & Weather forecasting app
- **Solution**: A smart bicycle that integrates with weather apps to suggest optimal routes and times to cycle, avoiding harsh weather conditions.

1. **Goal**: Help create daily routines that ensure productivity.

- **Items**: Coffee machine & Task management software
- **Solution**: A coffee machine that syncs with your task manager. Only when you complete certain morning tasks will it brew your coffee, incentivizing a productive start to the day.

**Easy Questions:**

1. **Goal**: Enable access to quality healthcare information.

- **Items**: Smartphone & Medical Journal
- **Solution**: An app that aggregates and simplifies articles from reputable medical journals, presenting them in a layperson-friendly format on the smartphone.

1. **Goal**: Enhance home security.

- **Items**: Webcam & Motion Detector Lights
- **Solution**: A motion-detector light system integrated with a webcam that begins recording when the light is triggered, storing footage securely for review.

1. **Goal**: Ensure personal digital privacy. (protect personal digital privacy)

- **Items**: Padlock & Web Browser
- **Solution**: A web browser with a built-in physical lock mechanism; only when the padlock is opened (using a unique key) can browsing history or saved passwords be accessed.

1. **Goal**: Aid in setting and tracking personal goals.

- **Items**: Calendar App & Pedometer
- **Solution**: A calendar app that integrates with a pedometer, allowing users to set and track daily step goals alongside other tasks and commitments.

1. **Goal**: Simplify grocery shopping.

- **Items**: Shopping cart & Barcode Scanner
- **Solution**: A shopping cart with a built-in barcode scanner that adds up prices as items are placed inside, providing a running total and helping to budget on the go.

**Moderate Questions:**

1. **Goal**: Encourage regular health check-ups.

- **Items**: Wristwatch & Blood Pressure Monitor
- **Solution**: A wristwatch that has an integrated blood pressure monitor, reminding users at regular intervals to check and log their blood pressure.

1. **Goal**: Provide tools for effective parenting.

- **Items**: Baby monitor & Parenting Blog
- **Solution**: A baby monitor that offers real-time parenting tips based on observed behaviors, sourced from reputable parenting blogs.

1. **Goal**: Boost motivation for personal goals.

- **Items**: Photo Frame & Fitness App
- **Solution**: A digital photo frame that syncs with a fitness app, displaying motivational pictures or quotes when certain milestones are achieved.

1. **Goal**: Foster personal creativity and expression.

- **Items**: Digital Camera & Coloring Book
- **Solution**: A digital camera that, when a picture is taken, turns the photo into a coloring book page, allowing users to creatively color their own captured moments.

1. **Goal**: Promote relaxation and mental breaks.

- **Items**: Hammock & Meditation App
- **Solution**: A hammock with built-in speakers that connect to a meditation app, offering guided relaxation sessions as you sway.

**Difficult Questions:**

1. **Goal**: Improve indoor air quality.

- **Items**: Window Blinds & Air Purifier
- **Solution**: Window blinds integrated with an air purifying system; as they're adjusted, they filter incoming air, ensuring better air quality indoors.

1. **Goal**: Facilitate better posture during work or study.

- **Items**: Office Chair & Virtual Reality (VR) headset
- **Solution**: An office chair that syncs with a VR headset, providing immersive environments that guide users into maintaining proper posture while they work or study in VR.

1. **Goal**: Enable easier access to books or learning materials.

- **Items**: Elevator & E-Library
- **Solution**: An elevator with built-in screens that access an e-library, allowing passengers to select and send books to their personal devices during their ride.

1. **Goal**: Encourage regular hydration (water consumption).

- **Items**: Desk Lamp & Water Filter
- **Solution**: A desk lamp that changes its color or dims based on when you last refilled your water from an integrated filter, reminding users to hydrate.

1. **Goal**: Facilitate household repair and maintenance.

- **Items**: Toolbox & Augmented Reality (AR) Glasses
- **Solution**: AR glasses that, when paired with a toolbox, overlay instructional guides on real-world items, assisting users in repairs and maintenance tasks.

**Easy Questions:**

1. **Goal**: Encourage safe online interactions.

- **Items**: Firewall & Social Media Platform
- **Solution**: A social media platform with a built-in firewall that scans and flags potentially harmful external links and profiles, ensuring safer interactions.

1. **Goal**: Enhance personal wardrobe and grooming (Improve your fashion choices and grooming routine).

- **Items**: GPS Device & Wardrobe Closet
- **Solution**: A wardrobe closet that uses GPS data to suggest outfits based on current weather and planned locations for the day.

1. **Goal**: Improve personal space organization.

- **Items**: Filing Cabinet & Smartphone Reminder App
- **Solution**: A filing cabinet with integrated smartphone reminders. When a document is placed inside, the system prompts the user to set a reminder related to the document.

1. **Goal**: Enhance pet care (improve pet care).

- **Items**: Food Dispenser & Webcam
- **Solution**: A pet food dispenser with an integrated webcam. Pet owners can monitor their pets and release food remotely through an app.

1. **Goal**: Simplify personal budgeting and saving.

- **Items**: Piggy Bank & Spreadsheet Software
- **Solution**: A digital piggy bank that automatically updates a spreadsheet with amounts saved, allowing users to track and analyze savings more efficiently.

**Moderate Questions:**

1. **Goal**: Enhance personal digital literacy.

- **Items**: E-book Reader & Typing Software
- **Solution**: An e-book reader with built-in typing software tutorials. As users read, they're occasionally prompted with interactive typing lessons related to the content.

1. **Goal**: Encourage environmental conservation at an individual level.

- **Items**: Bicycle & Recycling App
- **Solution**: A bicycle with an integrated app that offers rewards or points for distances cycled. Points can be redeemed for discounts on eco-friendly products.

1. **Goal**: Boost resilience and coping mechanisms (Strengthen one's capacity to endure and manage difficulties).

- **Items**: Stress Ball & Meditation App
- **Solution**: A stress ball that syncs with a meditation app. When squeezed, the app initiates a quick calming exercise or meditation to help with immediate stress relief.

1. **Goal**: Improve (boost) personal energy levels.

- **Items**: Coffee Machine & Fitness Tracker
- **Solution**: A coffee machine that adjusts brew strength based on sleep and activity data from a synced fitness tracker, providing an energy boost tailored to the user's needs.

1. **Goal**: Ensure a safe online shopping experience.

- **Items**: Digital Wallet & Review Platform
- **Solution**: A digital wallet integrated with a review platform, ensuring payments are only released to highly-rated and verified sellers.

**Difficult Questions:**

1. **Goal**: Encourage regular dental check-ups.

- **Items**: Electric Toothbrush & Calendar Software
- **Solution**: An electric toothbrush with integrated calendar reminders. After a set period, the toothbrush reminds users to schedule their next dental check-up.

1. **Goal**: Enhance personal communication skills.

- **Items**: Voice Recorder & Language Learning App
- **Solution**: A voice recorder that integrates with a language app, allowing users to practice and review their pronunciation and conversational skills.

1. **Goal**: Assist in managing dietary restrictions.

- **Items**: Refrigerator & Allergy Alert App
- **Solution**: A refrigerator with an integrated app that scans barcodes of stored items and alerts users if any contain allergens or ingredients they've listed as restricted.

1. **Goal**: Encourage positive community engagement.

- **Items**: Public Park Bench & Social Networking Site
- **Solution**: A public park bench with built-in connectivity to a local community's social networking site, encouraging users to engage in real-time events or activities happening nearby.

1. **Goal**: Aid in the care of elderly family members.

- **Items**: Walking Stick & Video Calling Device
- **Solution**: A walking stick with an integrated video-calling device, allowing elderly users to instantly connect with family or caregivers if they need assistance.

**Easy Questions:**

1. **Goal**: Strengthen personal relationship bonds.

- **Items**: Digital Photo Frame & Messaging App
- **Solution**: A digital photo frame that displays pictures sent via a messaging app, allowing loved ones to easily share moments even when apart.

1. **Goal**: Enhance indoor lighting.

- **Items**: Solar Panel & Mood Lamp
- **Solution**: A mood lamp powered by a portable solar panel. It adjusts lighting based on collected solar energy, providing both eco-friendly and mood-enhancing light.

1. **Goal**: Improve local travel experiences (Make local trips more enjoyable and satisfying).

- **Items**: Bicycle & GPS Navigation App
- **Solution**: A bicycle with an integrated GPS app that suggests scenic or less-traveled routes, ensuring a unique local travel experience.

1. **Goal**: Simplify home gardening and plant care.

- **Items**: Drip Irrigation System & Plant Care App
- **Solution**: A drip irrigation system that syncs with a plant care app, providing optimal watering schedules and care tips for specific plants.

1. **Goal**: Enhance personal entertainment experiences.

- **Items**: Virtual Reality Headset & Music Streaming Service
- **Solution**: A VR headset integrated with a music streaming service, providing immersive visual concerts or music videos.

**Moderate Questions:**

1. **Goal**: Improve home energy efficiency (To improve efficiency of home energy usage).

- **Items**: Smart Thermostat & Energy Monitoring Device
- **Solution**: A smart thermostat that works in tandem with an energy monitoring device, adjusting home temperatures in real-time based on energy consumption patterns.

1. **Goal**: Foster a better work-life balance.

- **Items**: Calendar Software & Fitness Tracker
- **Solution**: Calendar software that syncs with a fitness tracker, prompting breaks or physical activities based on scheduled work hours and sedentary periods.

1. **Goal**: Encourage continuous self-growth.

- **Items**: E-book Reader & Goal-setting App
- **Solution**: An e-book reader integrated with a goal-setting app. As users read self-help or educational books, they can set actionable goals directly on the device.

1. **Goal**: Foster intergenerational bonding within families. (cross-generational)

- **Items**: Board Game & Video Calling Device
- **Solution**: A board game that integrates with a video calling device, allowing family members from different locations to play together in real-time.

1. **Goal**: Strengthen immune system health.

- **Items**: Blender & Health and Nutrition Database
- **Solution**: A blender that syncs with a health and nutrition database, suggesting smoothie recipes tailored to boost specific vitamins or nutrients.

**Difficult Questions:**

1. **Goal**: Enhance personal spiritual or meditation practices.

- **Items**: Aromatherapy Diffuser & Meditation Music Streaming Service
- **Solution**: An aromatherapy diffuser integrated with a meditation music streaming service, providing a synchronized sensory experience to enhance meditation.

1. **Goal**: Encourage sustainable personal shopping habits (Promote eco-friendly individual shopping practices).

- **Items**: Barcode Scanner & Sustainability Rating App
- **Solution**: A handheld barcode scanner that, when linked to a sustainability rating app, instantly provides eco-friendly ratings or alternatives for products.

1. **Goal**: Improve the comfort of personal living spaces.

- **Items**: Recliner Chair & Ambient Noise Machine
- **Solution**: A recliner chair with an integrated ambient noise machine, allowing users to relax in a comfortable position while listening to calming sounds.

1. **Goal**: Aid in personal crisis management.

- **Items**: First Aid Kit & Crisis Helpline App
- **Solution**: A first aid kit with a built-in interface for a crisis helpline app, offering immediate assistance or guidance during emergencies.

1. **Goal**: Encourage self-reflection and mindfulness.

- **Items**: Journal (Dairy Book) & Biofeedback Device
- **Solution**: A journal integrated with a biofeedback device. As users write, they receive real-time feedback on their emotional state, encouraging more mindful reflection.

**Easy Questions:**

1. **Goal**: Develop methods to upcycle waste.

- **Items**: 3D Printer & Old Tires
- **Solution**: Use a 3D printer to reshape shredded old tires into sustainable furniture or home decor.

1. **Goal**: Foster gender equality in workplaces.

- **Items**: Job Recruitment Software & Board Game
- **Solution**: A job recruitment platform that uses mechanics from board games, where applicants' gender is hidden and skills are gamified, ensuring unbiased hiring.

1. **Goal**: Enhance potable water purification methods (Develop more effective ways to purify water for human consumption).

- **Items**: Coffee Maker & Bamboo Charcoal
- **Solution**: Modify a coffee maker to use bamboo charcoal filtering, making it a potable water purification device.

1. **Goal**: Encourage reforestation efforts (facilitate the restoration of forests).

- **Items**: Drone & Seed Pellets
- **Solution**: Use drones to disperse seed pellets over deforested areas, enabling efficient large-scale reforestation.

1. **Goal**: Design tools for monitoring air quality.

- **Items**: Smartwatch & Smoke Detector
- **Solution**: Integrate a smoke detector's air quality sensing capabilities into a smartwatch, allowing individuals to monitor the air quality wherever they go.

**Moderate Questions:**

1. **Goal**: Design systems for clean energy generation in urban settings.

- **Items**: Exercise Gym Equipment & Solar Panels
- **Solution**: Retrofit gym equipment to convert kinetic energy into electrical energy, then store and supplement this energy with solar panels.

1. **Goal**: Promote sustainable fishing practices.

- **Items**: Fish Finder Sonar & Barcode System
- **Solution**: Equip fish finder sonars with a barcode system that tags and tracks sustainable fish populations, ensuring fishermen target sustainable sources.

1. **Goal**: Design affordable housing with minimal environmental impact.

- **Items**: Shipping Containers & Green Roof Systems
- **Solution**: Transform shipping containers into living spaces and equip them with green roof systems for natural insulation and vegetation.

1. **Goal**: Improve public health in low-income areas.

- **Items**: Mobile Clinic & Bicycle
- **Solution**: Develop bicycle-powered mobile clinics that can easily navigate through congested areas, providing basic medical services to residents.

1. **Goal**: Encourage sustainable tourism (Advocate for eco-friendly travel options).

- **Items**: Virtual Reality Goggles & Travel Blogging Platform
- **Solution**: Use VR goggles in conjunction with travel blogging platforms to offer virtual tours, minimizing the carbon footprint of travel.

**Difficult Questions:**

1. **Goal**: Improve education access in remote areas.

- **Items**: Satellite Internet Service & E-Ink Tablets
- **Solution**: Use e-ink tablets connected via satellite internet services to provide educational resources and interactive lessons to students in remote areas.

1. **Goal**: Foster sustainable farming practices.

- **Items**: Automated Tractor & Worm Farm
- **Solution**: Equip automated tractors with worm farm systems, so while the land is tilled, it's simultaneously enriched with worm castings, promoting organic farming.

1. **Goal**: Promote community-based renewable energy projects.

- **Items**: Wind Turbine Kits & Crowdfunding Platform
- **Solution**: Sell DIY wind turbine kits on a crowdfunding platform, allowing communities to collectively fund and build their own renewable energy sources.

1. **Goal**: Develop efficient public transportation systems.

- **Items**: Maglev Train Technology & Carpool App
- **Solution**: Develop a maglev-based public transport system with routes based on real-time data from carpool apps, ensuring efficient routes and minimal congestion.

1. **Goal**: Create awareness about responsible consumption (Make people more aware of the impacts of their consumption choices).

- **Items**: Augmented Reality Glasses & Shopping Scanner
- **Solution**: Design AR glasses that, when synced with a shopping scanner, overlay information about products' sustainability, carbon footprint, and ethical sourcing, educating shoppers in real-time.

**Easy Questions:**

1. **Goal**: Promote gender equality in political representation.

- **Items**: Social Media Platform & Chess Set
- **Solution**: Create a social media campaign using the theme of a chess set, where every piece (irrespective of its traditional "power" or role) has equal importance, emphasizing the importance of diverse representation in politics.

1. **Goal**: Foster innovation in sustainable packaging (Propose new ideas for packaging that is both innovative and eco-friendly).

- **Items**: Beeswax & Popsicle Sticks
- **Solution**: Design a biodegradable packaging solution using molded beeswax reinforced with popsicle stick frames.

1. **Goal**: Promote equal access to technology in underserved communities.

- **Items**: Library System & Refurbished Laptops
- **Solution**: Introduce a laptop-lending program in libraries where refurbished laptops are loaned out to community members, ensuring technological access to all.

1. **Goal**: Foster education on climate change.

- **Items**: Animated Series (Animation production) & Weather Station Data
- **Solution**: Produce an animated series that uses real data from weather stations to teach children about the impacts and science of climate change.

1. **Goal**: Encourage sustainable fashion (Advocate for clothing that's better for the environment).

- **Items**: Bamboo Fiber & QR Code
- **Solution**: Create clothing from bamboo fiber that includes a QR code. When scanned, the code provides information about the sustainable process used in making the item, educating consumers.

**Moderate Questions:**

1. **Goal**: Design water conservation systems for agriculture.

- **Items**: Drip Irrigation System & Rainwater Harvesting Kits
- **Solution**: Combine drip irrigation with rainwater harvesting kits, using collected rainwater for precise irrigation, reducing water wastage.

1. **Goal**: Improve disaster preparedness in vulnerable communities.

- **Items**: Mobile Alert App & Community Radio Station
- **Solution**: Integrate a mobile alert system with community radio stations, ensuring timely disaster warnings and safety guidelines are broadcasted and received by all, including those without smartphones.

1. **Goal**: Create sustainable urban green spaces.

- **Items**: Rooftop Gardens & Public Transport Stations
- **Solution**: Design public transport stations with rooftop gardens, providing both transportation and green spaces in urban settings.

1. **Goal**: Encourage biodiversity conservation.

- **Items**: Wildlife Documentary & Augmented Reality (AR) App
- **Solution**: Create an AR app that works in tandem with wildlife documentaries. Viewers can virtually "interact" with the species they're learning about, fostering a deeper appreciation and understanding.

1. **Goal**: Promote peacebuilding in conflict zones.

- **Items**: Music Festivals & Virtual Reality (VR) Headsets
- **Solution**: Organize virtual reality music festivals where people from conflict zones can participate, enjoy, and interact in a peaceful digital environment, fostering unity and understanding.

**Difficult Questions:**

1. **Goal**: Develop efficient waste management systems.

- **Items**: Conveyor Belt System & Artificial Intelligence (AI) Sorting Algorithm
- **Solution**: Implement a waste management system where conveyor belts transport waste, and an AI algorithm identifies and sorts them for recycling or disposal.

1. **Goal**: Improve sanitation facilities in underserved areas.

- **Items**: Biodegradable Soaps & Bicycle-Powered Water Pumps
- **Solution**: Distribute biodegradable soaps along with bicycle-powered water pumps in areas lacking sanitation facilities. The bikes pump and provide clean water while promoting hygiene with the soap.

1. **Goal**: Foster community-based health initiatives (promote health projects driven by and for the community).

- **Items**: Fitness Tracker & Local Farmer's Market
- **Solution**: Collaborate with local farmer's markets to sync data from fitness trackers, providing discounts or rewards for healthy activities, thus encouraging community health.

1. **Goal**: Design products using sustainable materials.

- **Items**: Algae-Based Plastics & Smart Home Devices
- **Solution**: Produce smart home devices using algae-based biodegradable plastics, promoting eco-friendly tech products.

1. **Goal**: Strengthen institutions for corruption prevention.

- **Items**: Blockchain Technology & Whistleblower Hotline
- **Solution**: Develop a whistleblower hotline system that logs reports on a transparent and tamper-proof blockchain, ensuring credibility and trust in anti-corruption efforts.

**Easy Questions:**

1. **Goal**: Design products to reduce carbon footprint (carbon dioxide emission).

- **Items**: Solar Panels & Smartphone Cases
- **Solution**: Design smartphone cases with embedded solar panels. The case charges the phone when exposed to sunlight, reducing the need for electric charging and thereby reducing carbon emissions.

1. **Goal**: Encourage youth involvement in sustainability projects.

- **Items**: Virtual Reality (VR) Games & School Curriculum
- **Solution**: Integrate a VR game into the school curriculum that allows students to build and manage sustainable cities, sparking interest in real-world sustainability projects.

1. **Goal**: Enhance public awareness on marine conservation.

- **Items**: Aquariums & Augmented Reality (AR) Apps
- **Solution**: Develop an AR app for aquarium visitors that overlays information about the impacts of pollution, overfishing, and climate change on displayed marine life.

1. **Goal**: Foster skills training for sustainable job opportunities (Promote the development of skills for long-term, sustainable employment).

- **Items**: Online Courses & Hand-Cranked Generators
- **Solution**: Offer online courses in sustainable job sectors that can be accessed in areas without reliable electricity using hand-cranked generators to power devices.

1. **Goal**: Design affordable, sustainable energy storage solutions.

- **Items**: Kinetic Energy & Old Car Tires
- **Solution**: Design a system where kinetic energy (from activities like walking or cycling) is stored in revamped old car tires converted into flywheels.

**Moderate Questions:**

1. **Goal**: Encourage partnerships for sustainability between countries.

- **Items**: International Film Festivals & Green Technologies (Environment-friendly)
- **Solution**: Organize an international film festival showcasing documentaries on green technologies from around the world, fostering collaboration and partnerships between countries.

1. **Goal**: Enhance waste reduction in food production (Develop effective approaches to reducing waste in food production).

- **Items**: Drones & Greenhouses
- **Solution**: Utilize drones in greenhouses to monitor and assess the health of crops, ensuring that any potential issues are addressed early on, minimizing waste.

1. **Goal**: Develop tools for monitoring water quality.

- **Items**: Smart Watches & Aquatic Plants
- **Solution**: Create smartwatches integrated with sensors that detect changes in aquatic plants, indicating changes in water quality.

1. **Goal**: Promote sustainable livestock farming (develop approaches for eco-friendly livestock farming).

- **Items**: Vertical Farms & Livestock Feed Apps
- **Solution**: Introduce vertical farming techniques for producing livestock feed and pair it with an app that calculates the optimal feed amount, reducing waste and ensuring efficient land use.

1. **Goal**: Encourage equal pay for equal work.

- **Items**: Cryptocurrencies & Job Portals
- **Solution**: Develop job portals where payment is made in transparent cryptocurrencies, making salary amounts and pay scales clear and promoting equal pay.

**Difficult Questions:**

1. **Goal**: Foster community resilience to climate change.

- **Items**: Community Theaters & Climate Models
- **Solution**: Use community theaters to showcase plays based on future climate models, helping communities visualize and prepare for potential challenges.

1. **Goal**: Promote conservation of freshwater resources.

- **Items**: Smart Mirrors & Evaporation Data
- **Solution**: Design smart mirrors for households that display daily evaporation data, reminding users to conserve water.

1. **Goal**: Encourage local sustainable product manufacturing (To encourage eco-friendly product manufacturing in local community).

- **Items**: 3D Printers & Local Artisanal Crafts
- **Solution**: Combine 3D printing technology with local artisanal designs to create sustainable products that also support local craftsmanship.

1. **Goal**: Foster inclusive education for differently-abled individuals.

- **Items**: E-books & Sign Language Algorithms
- **Solution**: Develop e-books integrated with sign language algorithms, providing real-time sign language interpretations for differently-abled readers.

1. **Goal**: Design infrastructure for efficient rainwater harvesting (Create a rainwater collection system for effective water storage).

- **Items**: Parking Lots & Ceramic Filters
- **Solution**: Retrofit parking lots with systems that collect rainwater, which then gets purified using ceramic filters, providing an urban solution to water scarcity.

**Easy Questions:**

1. **Goal**: Enhance digital literacy in underserved communities.

- **Items**: E-readers & Solar-powered lanterns
- **Solution**: Provide e-readers with pre-loaded digital literacy tutorials that are powered by integrated solar lanterns to communities without regular electricity access.

1. **Goal**: Design tools for early warning systems for natural disasters.

- **Items**: Weather Apps & Doorbells with cameras
- **Solution**: Upgrade doorbell cameras to detect unusual environmental changes (e.g., rapid cloud formations, increased wind speed) and sync with a weather app to provide real-time disaster warnings to residents.

1. **Goal**: Promote sustainable urban transportation.

- **Items**: Bicycles & Ride-sharing Apps
- **Solution**: Introduce a bicycle-sharing feature in existing ride-sharing apps, promoting eco-friendly transportation in urban areas.

1. **Goal**: Enhance local food production in urban areas.

- **Items**: Rooftops & Hydroponic systems (Water-based gardening systems)
- **Solution**: Develop easily-installable hydroponic systems for urban rooftops, turning them into mini-farms and promoting local food production.

1. **Goal**: Design sustainable solutions for electronic waste.

- **Items**: Vending Machines & Recycling Apps
- **Solution**: Create vending machines where users can drop off electronic waste. The machine scans the item, credits the user's recycling app, and ensures the waste is sent to appropriate recycling facilities.

**Moderate Questions:**

1. **Goal**: Foster child rights and child protection measures.

- **Items**: Wearable GPS Trackers & Educational cartoons
- **Solution**: Produce educational cartoons that come with a wearable GPS tracker for children, teaching them about safety and child rights while providing parents a way to locate their kids.

1. **Goal**: Promote eco-friendly travel.

- **Items**: Electric Cars & Travel Review Websites
- **Solution**: Integrate an eco-friendly rating system into travel review websites, giving higher scores to destinations accessible by electric cars, promoting green travel.

1. **Goal**: Enhance public awareness on land conservation.

- **Items**: Digital Billboards & Drones
- **Solution**: Use drones to capture real-time footage of deforestation or land degradation and display it on digital billboards in urban areas, increasing awareness.

1. **Goal**: Encourage fair trade practices.

- **Items**: QR Codes & Coffee Machines
- **Solution**: Equip coffee machines to only accept coffee brands with QR codes certifying fair trade practices, promoting ethical consumption.

1. **Goal**: Develop solutions to combat desertification (develop solutions to prevent desertification (land turning into desert-like conditions)).

- **Items**: Aerial Seed Bombing & Moisture Harvesters
- **Solution**: Use aerial seed bombing techniques in conjunction with moisture harvesters to reintroduce vegetation to desertified areas.

**Difficult Questions:**

1. **Goal**: Design methods for carbon capture and storage.

- **Items**: Algae & Underground Metro Systems
- **Solution**: Design metro station infrastructure to integrate algae-based systems that capture carbon from the air, then store the captured carbon in underground chambers.

1. **Goal**: Foster economic growth using sustainable practices.

- **Items**: Crowdfunding Platforms & Green Tech Start-ups
- **Solution**: Create a dedicated crowdfunding platform for green tech start-ups, ensuring they receive the funding to grow while promoting sustainable practices.

1. **Goal**: Enhance public health through clean air initiatives.

- **Items**: Air Purifiers & Public Buses
- **Solution**: Integrate large-scale air purifiers into public bus systems, cleaning the air as they move through urban areas.

1. **Goal**: Foster community-based wildlife conservation (Encourage community involvement in preserving wildlife).

- **Items**: Community Radios & Wildlife Cameras
- **Solution**: Set up community radios that broadcast real-time footage from wildlife cameras, engaging locals in immediate conservation efforts.

1. **Goal**: Encourage partnerships for technology transfer in sustainability (Promote collaborations to share sustainable technology).

- **Items**: Virtual Reality Conferences & University Research Labs
- **Solution**: Organize virtual reality conferences where university research labs showcase their latest sustainable technologies, fostering global collaborations and technology transfers.
